# The CH1α domain of mucosal gp41 IgA contributes to antibody specificity and antiviral functions in HIV-1 highly exposed Sero-Negative individuals

**DOI:** 10.1371/journal.ppat.1009103

**Published:** 2020-12-14

**Authors:** Marwa Khamassi, Lin Xu, Julien Rey, Maxence Duchemin, Tahar Bouceba, Pierre Tuffery, Daniela Tudor, Morgane Bomsel

**Affiliations:** 1 Laboratory of Mucosal Entry of HIV-1 and Mucosal Immunity, Department of Infection, Immunity and Inflammation, Cochin Institute, CNRS UMR 8104, Paris, France; 2 INSERM U1016, Paris, France; 3 Université Paris, Paris, France; 4 Unité de Biologie Fonctionnelle et Adaptative - CNRS UMR 8251 - INSERM ERL U1133, Ressources Parisienne en Bioinformatique Structural, Paris, France; 5 Sorbonne University, CNRS, Institut de Biologie Paris-Seine (IBPS), Protein engineering platform, Molecular Interaction service, Paris, France; Emory University, UNITED STATES

## Abstract

The antibody molecule comprises a variable domain conferring antigen specificity and affinity distinct from the heavy chain constant (CH) domains dictating effector functions. We here interrogate this paradigm by evaluating the unique influence of the CH1**α** domain on epitope specificity and functions using two mucosal gp41-specific Fab-IgAs (FabA) derived from HIV-1 highly-exposed but persistently seronegative individuals (HESN). These HESN develop selectively affinity-matured HIV-1-specific mucosal IgA that target the gp41 viral envelope and might provide protection although by unclear mechanisms. Isotype-switching FabAs into Fab-IgGs (FabGs) results in a >10-fold loss in affinity for HIV-1 clade A, B, and C gp41, together with reduced neutralization of HIV-1 cross-clade. The FabA conformational epitopes map selectively on gp41 in 6-Helix bundle and pre-fusion conformations cross-clade, unlike FabGs. Finally, we designed *in silico*, a 12 amino-acid peptide recapitulating one FabA conformational epitope that inhibits the FabA binding to gp41 cross-clade and its neutralizing activity. Altogether, our results reveal that the CH1**α** domain shapes the antibody paratope through an allosteric effect, thereby strengthening the antibody specificity and functional activities. Further, they clarify the mechanisms by which these HESN IgAs might confer protection against HIV-1-sexual acquisition. The IgA-specific epitope we characterized by reverse vaccinology could help designing a mucosal HIV-1 vaccine.

## Author summary

In HIV-1 highly exposed persistently seronegative individuals (HESN), protection is a multifactorial phenomenon whereby mucosal IgA, but not IgG, play a determinant anti-viral role. The HESN IgA response focuses on conserved regions of the HIV-1 envelope gp41 subunit, in contrast with the broader IgG and IgA response observed in HIV-1-infected individuals. How protective only IgA, but not IgG, develop in HESN remains unclear. We addressed the role of the CH1 domain of each isotype in antigen recognition and functions by comparing mucosal gp41 specific Fab-IgA1 (FabA) derived from HESN with paired Fab-IgG1 (FabG). We show that structural differences in the CH1α and γ domains impact affinity constants, FabA having higher affinity for gp41 derived from clade A, B and C. FabA neutralization of clade A, B and C HIV-1 infection and transfer from Langerhans to CD4+T cells were cross clade and higher than that observed with corresponding IgG. Accordingly, FabA and G recognize different gp41 epitopes on gp41 clade A, B and C gp41 crystal structures. Using molecular modeling, we defined a peptide corresponding to one 3-D epitope common to gp41 clade A, B and C gp41 specific for one of the FabA that reverses the FabA neutralizing activities. Altogether, we show here that the CH1 domain influences the antibody paratope and in turn its specificity and functional activities. The FabA cross-clade epitopes retrieved on gp41 could be useful in mucosal vaccine design against HIV-1.

## Introduction

HIV-1 infection is mainly initiated at mucosal sites during sexual intercourse, and IgA can efficiently prevent HIV-1 infection and mucosal reservoir establishment (reviewed in [1–4]). We and others have previously shown that mucosal IgAs, specific for HIV-1 envelope gp41-subunit conserved epitopes, occur naturally in 1% of individuals subsequent to sexual exposure to HIV-1, who remain persistently uninfected. These individuals, referred to as Exposed SeroNegative (HESN), remain IgG seronegative but raise gp41-specific mucosal IgAs that constitute a main correlate of protection (reviewed in [3–5]). *In vitro*, HESN IgAs are able to inhibit HIV-1 mucosal penetration by transcytosis across epithelial barriers and to neutralize CD4^+^T-cell infection against clade B HIV-1 [3,6,7].

Remarkably, at the molecular level, we have shown that HESN IgAs are affinity-matured with extensive somatic hypermutations, and have extended heavy-chain third complementarity determining regions (CDRH3) [3], characteristics similar to those found for IgGs in natural HIV-1 infection, and that increase breadth and potency *in vitro* [8–10]. Compared to HIV-1 infected patients IgGs, mucosal HESN IgAs are generated earlier after HIV-1 exposure and by interfering with mucosal virus translocation might participate in HESN individual protection from infection [4,11]. The presence of HESN-protective gp41-specific IgAs at the mucosal level, affords evidence for an efficient “natural” vaccination. Furthermore, this IgA-restricted immune response in HESN raises questions about the protective mechanisms of a humoral response involving only mucosal IgA, and, at the molecular level, about the participation of the isotype-specific constant regions of the antibody in the efficacy of HESN IgAs.

Antibodies (Ab) have been historically described as being composed of two heavy and two light chains, each one consisting of a variable and a constant region. The variable regions of both chains form the paratope that is responsible for antigen binding, and the heavy chain constant regions mediates cellular effector functions such as antibody-dependent cell cytotoxicity (ADCC) and antibody-dependent phagocytosis (ADCP). Recent works including our own [12], revealed that the Ab isotype carried by the entire Ab heavy chain constant region, containing the constant heavy chain (CH)1, CH2 and CH3, influences the paratope structure and in turn the antigen recognition (reviewed in [13]). In particular, the broadly neutralizing antibody (bNAb) 2F5 IgG1, specific for gp41, derived from the blood of an HIV-1-infected patient [14] was isotype-switched into an IgA2 allowing to compare the role of the constant alpha and gamma regions in antibody structure/function. As a result, we showed that the three CH alpha domains together, which form the IgA constant heavy chain, improve 2F5 IgA affinity and, in turn, antiviral functions compared to that of 2F5 IgG [12]. Changes induced by isotype-switching are accompanied by modulation of epitope specificity, and most probably occur by change in paratope conformation due to non-local [15] and even allosteric effects that modify Ab-antigen interaction [16], antigen binding impacting the constant region and *vice versa* [17].

Which CH domain(s), among CH1, CH2 and CH3, participate(s) in modulation of Ab specificity and affinity remains unclear. The CH1, separated from the CH2 and CH3 by the hinge region, belongs to the Fab region of the Ab and is immediately juxtaposed to the paratope formed by the variable heavy (VH) and light (VL) chains. The CH2 and CH3 are known to participate in Fc-receptor binding on effector cells to mediate ADCC or ADCP. Whether the CH1 itself, due to its proximity to the VH and VL regions, plays a determinant role in Ab affinity and functions, independently of the CH2 and CH3 has not been studied.

Furthermore, the role of constant regions has only been evaluated by comparing blood-derived antibodies expressing identical V regions in different heavy chain constant contexts and thus, addressing a blood generated B-cell epitope. However, mucosal immunity is highly compartmentalized [18]. Whether the Ab isotype also affects Ab specificity and affinity and even function of mucosal Abs in a similar manner than blood-derived Abs remains elusive.

Therefore, to specifically address the impact of the CH1 in Ab specificity and functional activities, we took advantage of two HESN mucosal anti-gp41 IgA1 in an Fab format (FabA) that we characterized previously [3], and isotype-switched into Fab-IgG1 (FabG). Each FabA and its respective isotype-switched FabG, were compared for HIV-1 envelope binding, epitope recognition, and anti-viral activities against the main HIV-1 strains present worldwide, namely HIV-1 of clades B, A and C, and their conformational epitopes on gp41 were determined using *in silico* analyses. These comparative analyses result in a molecular understanding of the recognition of different epitopes on the HIV-1 envelope, depending on the isotype, in correlation with different affinities and antiviral functions. Furthermore, the conformational epitope we determined for one of the FabA could be used as antigen in HIV-1 vaccine design.

## Results

### CH1 isotype-switching of mucosal FabA into FabG

To evaluate the role of the CH1 domain in mucosal Ab specificity and function, two FabA clones, namely 43 and 177, were studied. These FabA were obtained previously [3] by screening an HESN FabA library on the clade B gp41 membrane proximal extended region (MPER) P1 [19,20] or on recombinant clade B gp41 deleted for its MPER [3], respectively. At the molecular level, FabA 43 has a high degree of somatic mutations, a VH3 heavy chain germline origin similar to the bNAb IgG 10E8 [21], and a long CDRH3 of 22 residues, features characteristics of other bNAb IgGs [3]. In contrast, FabA 177 has a low level of somatic mutations in the heavy chain, 100% homology with germ-line gene region IGKVID-30*-01, and a normal-length CDRH3 of 11 residues [3]. Furthermore, the FabA 177 heavy chain originates from the VH6 family, a germline origin never associated to bNAb IgGs. Each FabA was transformed into the corresponding FabG by genetic engineering, replacing the CH1α1 by a CH1γ1, and purified by affinity chromatography, as described in the Material and Methods section. In each pair, isotypes share identical VH and VL domains and the same light chain, solely differing by the CH1 domain.

### FabA and FabG binding and affinity for HIV-1 envelope

**Fig 1:**
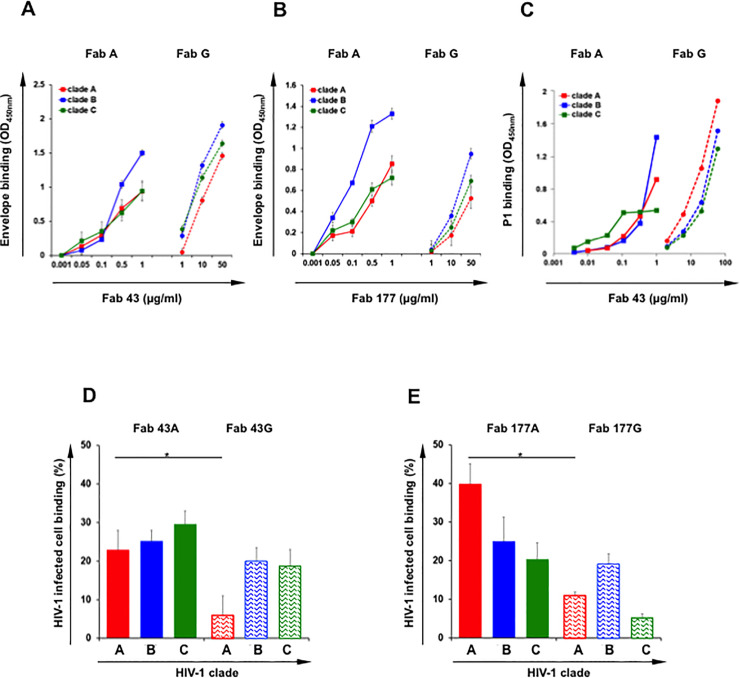
**FabA and G of clones 43 and 177 binding to HIV envelope gp41 clades A, B and C in a dose dependent manner**. **A and B**: The specificity of FabA (solid line) and FabG (dotted line) for clade A gp140 (red), clade B gp41 (blue) clade C gp41 (green) was evaluated by ELISA. For direct comparison of FabA and G isotypes, detection was performed using an anti-kappa light chain. Specific binding (OD450 nm) is plotted as a function of Fab concentration (μg/ml). Values represent mean ± SEM, derived from 3 independent experiments performed in duplicate. (**A)** Fab 43, (**B**) Fab 177. **C**: The specificity of FabA 43 (solid line) and FabG 43 (dotted line) for the peptide P1 cross clade, namely clade A (red), clade B (blue) and clade C (green) was evaluated by ELISA. One representative out of three independent experiments performed in duplicate is shown. **D and E:** Binding of FabA and G of clones 43 and 177 to HIV-1 infected **CD4**^**+**^**T-cells.** FabA (solid bars) and FabG (hatched bars) from clones 43 (**D**) and 177 (**E**) were incubated with Clade A (red) or clade B (blue) and clade C (green) HIV-infected cells or uninfected cells as negative control overnight at 4°C. Irrelevant IgA and IgG were used as negative controls. Specific binding was detected using FITC conjugated anti-human kappa light chain to allow for direct comparison of both FabA and G isotypes, and analyzed by flow cytometry, as indicated in Materials and methods section. Values represent the % of Fab+± SEM among HIV-1-infected (Gag-p24+) CD4+T-cells. Binding of all Fabs to non-infected cells was negligible. Binding of irrelevant IgA and IgG to infected cells it was fixed at 1% and subtracted from the specific binding values; n = 3 independent experiments. Student’s t–test, * = p<0.05.

We first evaluated whether class-switching preserves the original binding properties of IgA by comparing FabA and FabG binding to recombinant trimeric gp41 derived from HIV-1 clade B [20] and gp140 derived from HIV-1 clades A and C, the three main HIV-1 clades prevalent worldwide. To avoid bias introduced by different isotype-specific antibodies used for detection, binding to gp41 in ELISA was revealed using mouse anti-human kappa light chain [12]. FabA 43 and 177 recognize conformationally-conserved regions on clade B gp41 from both R5 and X4 tropic viruses [12]. We found that both FabA and G pairs specifically bind to clade B gp41 as well as to clades A and C gp140 in a dose-dependent manner, but strikingly for all clades, binding of FabA 43 and 177 is more efficient compared to that of their respective FabG (Fig 1A and 1B). Hence, a 50-fold higher concentration of FabG 43 (50μg/ml) is required to reach the same clade B gp41 binding of FabA 43 (1μg/ml) (Fig 1A). Differences observed between FabA and G 177 are even larger, reaching 500-fold between the isotypes in favor of FabA (Fig 1B). Similarly, binding to a constant quantity of clade A and C gp140, required a 10-fold less FabA 43 than FabG 43 (Fig 1A), and a 50-100-fold less FabA 177 than FabG 177 (Fig 1B), respectively. Fab-IgA D1.3 specific for lysozyme [3] and irrelevant Fab-IgG used as a negative control do not recognize none of either clade B gp41 or clade A and C gp140.

Next, surface plasmon resonance (SPR) was used in kinetic experiments to compare FabA and G affinity to clade B gp41, clade A or C gp140 immobilized on the sensor chip surface. FabA and FabG 43 and 177 at various concentrations, were the analytes. As a result (Table 1 and S1 Fig), when clade B gp41 serves as target, KD is 3.62 nM for FabA 43 and 21.2 nM for FabA 177 compared to 1.12 and 1.22 μM for their respective FabG. When clade A and C gp140 serve as target, KDs are 6.41 and 4.79 nM for the FabA 43 compared to 500 nM, and no affinity is measured for the paired FabG 43. KD equals 0.29 and 21.5 nM for FabA 177, whereas paired FabG 177 has no measurable affinity for either the clade A or C viral envelopes.

**Table 1:** FabA has greater affinity for gp140 clades A and C and for gp41 MPER clade B compared with FabG.

Ligand	Fab Isotype	KD (M)	
		Fab 43	Fab 177
**gp140** **clade A**	**IgA**	6.41x10^-9^	2.92x10^-10^
	**IgG**	5.05x10^-7^	no binding
**gp41** **clade B**	**IgA**	3.62x10^-9^	2.12x10^-8^
	**IgG**	1.12x10^-6^	1.22x10^-6^
**gp140** **clade C**	**IgA**	4.79x10^-9^	2.15x10^-8^
	**IgG**	no binding	no binding

Antigen was immobilized on a CM-5 chip for surface plasmon resonance evaluation of an antibody affinity constant for P1. FabA and FabG were the analytes. The KD values shown were estimated by global curve fitting of the specific binding responses from n = 2 independent experiments performed each at n = 4 to 8 analyte concentrations as described in the methods section. In all cases, corresponding Pearson’s χ2 test (Chi2) was <10 and considered statistically significant [12].

Overall, affinities for HIV envelopes of all clades reach the nM range for both FabA 43 and 177 clones, similar to that of other bNAbs [22], whereas it remain in the order of μM or even lower for the corresponding FabG, in agreement with binding experiments using ELISA (Fig 1). Importantly, for all Fabs taken together, affinity correlates with binding observed by ELISA (spearman r = 0.606; p = 0.0375)

### Binding of FabA and FabG to the P1 peptide

The 35 amino acid peptide P1 was characterized as the minimal membrane proximal external region of the viral envelope, gp41, that allows for HIV-1 binding to galactosyl ceramide, the HIV-1 mucosal receptor and for mucosal HIV-1 entry by transcytosis [23–25]. This highly conserved region has been used as immunogen in a prophylactic vaccine against HIV-1 both in pre-clinical and clinical phase I trials [20,26]. P1 sequence is well conserved between clade B and A viruses whereas a K670S mutation in clade C virus prevents binding of the bNAb 2F5 IgG [27]. FabA 43 was obtained by screening the FabA HESN library on clade B P1 and consequently FabA 43 binds to clade B P1 [3]. When studied by NMR, P1 3-Dimensional structure is not linear but contains 2 helices separated by a bent [28]. We thus evaluated by ELISA, the capacity of FabA and G 43 to bind to clade A, B and C P1, comparatively as described [3]. Both FabA and G 43 isotypes bind to clades A, B and C P1 in a dose-dependent manner (Fig 1C). Strikingly as above, when the P1 peptide is expressed in the context of a full protein (Fig 1A), FabA 43 recognizes P1 more efficiently than its corresponding FabG (Fig 1C). This difference is higher when clade B P1 is the target (with a 60-fold change from FabA to G), but still significant (with a 20-fold change from FabA to G) when clade A and C are targets (Fig 1C). Additionally, FabA and FabG 43 bind more efficiently to clade B compared to clade A and C P1 (Fig 1C). These differences in Fab isotype binding to P1 from the three clades are also observed by SPR (S2 Fig).

Overall, the FabA 43 binds the gp41 subunit P1 cross clade more efficiently than its FabG counterparts with an affinity in the nM range.

### FabA and FabG binding to HIV-infected CD4^+^ T-cells

**Fig 2:**
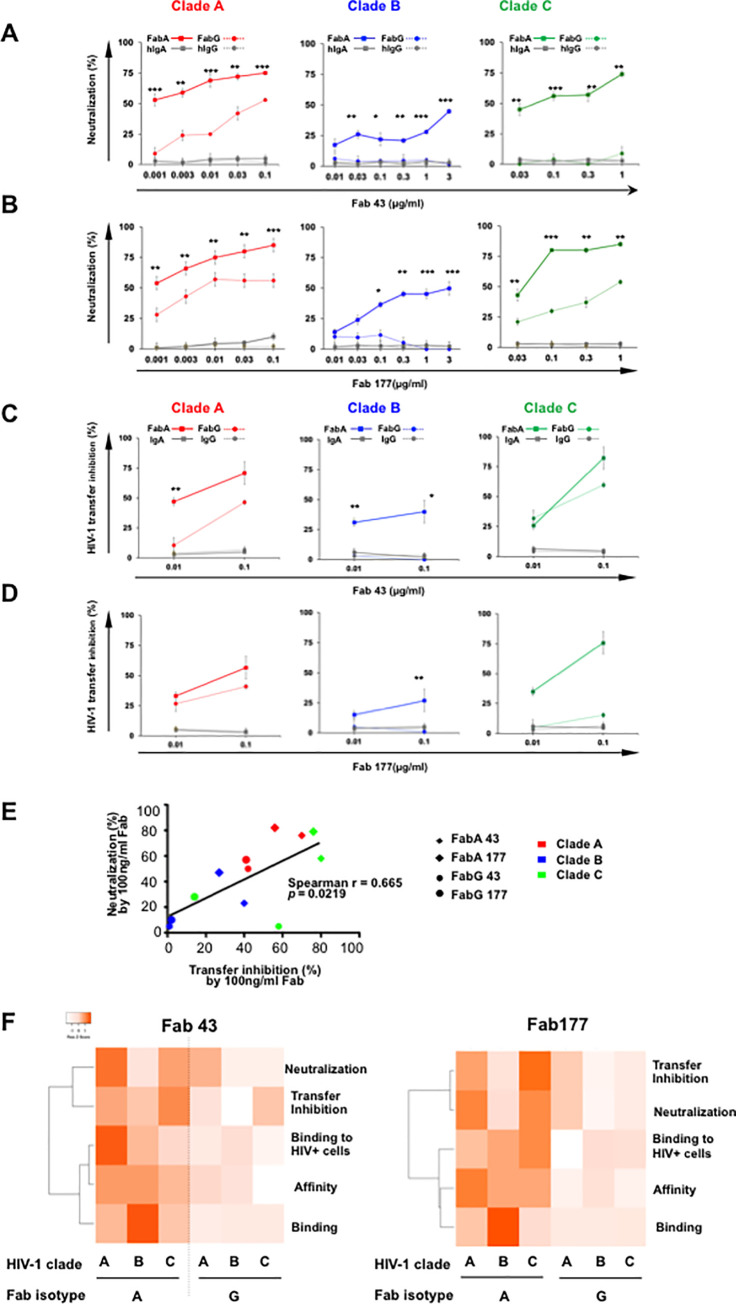
**Fab 43 and 177-A and -G antiviral efficacy against clades A, B and C HIV-1. (A** and **B**) Dose-dependent inhibition of HIV-1 infection mediated by Fab 43 (**A**) and Fab 177 (**B**) assessed in a single cycle infectivity assay, using p24 staining on CD4^+^T-cells. For each Fab clone, FabA (solid line), and FabG (dotted line), were incubated for 1 h at 37°C with either HIV-1 clade A (red), clade B (blue) or with clade C (green) before the addition of CD4^+^T cells for 36 h. Irrelevant Fab-IgA (solid grey line) and Fab-IgG (dotted grey line) at similar concentrations were used as negative controls. The percentage of neutralization, analyzed by flow cytometry as indicated in the Materials and methods section, was defined as the reduction of Gag-p24^+^ HIV-1-infected cells compared with HIV-1-infected cells in the absence of Fab. Values represent mean ± SEM, derived from at least 4 independent experiments performed in triplicate. Student’s t–test, * = p<0.01; ** = p<0.001; *** = p<0.00001. **(C** and **D**) Inhibition of HIV-1 transfer from LCs to autologous CD4^+^T cells evaluated by measuring the p24 released by LCs/T cell co-cultures at day 5. HIV-1 of either clade A (red), clade B (blue) or clade C (green) was pre-incubated with LCs for 2 h before addition of the Fabs (FabA solid line, FabG dotted line) and CD4^+^T-cells. Irrelevant Fab-IgA (solid grey line) and Fab-IgG (dotted grey line) at similar concentrations were used as negative controls. The LC–T cell cocultures were incubated at 37°C for 7d. LC–T cocultures without Fab were considered 100% HIV-1 transfer from LC to autologous CD4^+^T-cells. Virus transfer was evaluated by measuring p24-Ag by ELISA as described in the Material and Methods section. Results correspond to the % inhibition of transfer in the presence of Fabs. Values represent mean ± SEM of at least five independent experiments performed in triplicate. Student’s t–test, * = p<0.01**; = p<0.01. **(E) Correlation between HIV-1 neutralizing activity and transfer inhibition.** Neutralization and transfer inhibition measured for clone 43 and 177 FabA and G at 100ng/ml, were correlated. Spearman rank correlation r and corresponding values are indicated**. (F) Correlation between performance in each assay of each clone 43 and 177**. Ratio of values obtained in each indicated assay for paired FabA and G of each clone, were obtained and plotted as a heatmap with using the complete linkage clustering method and ranking according to Spearman statistics. Binding represents 1/HIV envelope ELISA OD values; Affinity corresponds to 1/log KD values.

We next evaluated the capacity of the Fabs to bind to the viral envelope in its trimeric spike conformation at the surface of CD4^+^T-cells infected with clade A (primary isolate 92UG031), B (isolate JR-CSF) or C virus (primary isolate 92BR025). After overnight incubation at 4°C with the various Fabs, binding was evaluated by flow cytometry using an anti-kappa light chain secondary antibody to allow direct isotype comparison. All Fabs bind specifically HIV-1 infected cells although binding of FabGs to clade C and clade A HIV-1 infected cells is much lower compared to FabAs. Between 20 and 40% of HIV-infected T-cells labeled for FabA compared to 5 and 19% for FabG (Student’s *t*–test, p<0.05) (Fig 1D and 1E). FabA 43 recognizes more efficiently clade A- than clade B- and C-infected CD4^+^T-cells (Fig 1D), whereas no differences were apparent between clades for FabA 177 (Fig 1E). Importantly, Fab binding to HIV-1-infected cells correlates with Fab affinity and binding to HIV-1 envelope measured by ELISA (Fig 1A–1C) (spearman r = 0.777 p = 0.0156) (Fig 2F).

### FabA and FabG antiviral properties

#### Neutralization of HIV-1 CD4^+^ T-cell infection by FabA and FabG

As FabA and G only differ by the CH1 domain, the results presented above suggest that the CH1 domain affects the paratope fitting on the antigen that might, in turn, impact the Ab anti-viral function in an isotype-dependent manner.

Therefore, we first investigated the impact of the CH1 isotype in Fab-neutralizing activities. Using a panel of three viruses from clade A, B and C, neutralization of CD4^+^T lymphocytes infection by both FabA and G pairs was tested as previously described [3]. Serial dilutions of each FabA and G were assessed comparatively, and IC50 values were determined for each HIV-1 clade. Comparative evaluation of paired Fab 43 isotypes reveals that FaA 43 neutralizes all three A, B and C clades with a respective IC50 of 1ng, 1μg and 100ng/ml, whereas FabG 43 only neutralizes clade A virus with an IC50 of 100ng/ml but not clades B or C virus (Fig 2A). A similar neutralization pattern is observed for the clone 177 with an IC50 of 1ng, 300ng and 30ng/ml for FabA 177 against clade A, B and C virus respectively and an IC50 of 100ng and 1μg for FabG 177 against clade A and C virus, respectively (Fig 2B). In all cases, the paired FabA has significantly-increased neutralization compared to its paired FabG for all concentrations evaluated (Student’s *t*–test, p values ranging from p<0.01 to p<0.00001). Irrelevant Fab-IgA and Fab-IgG, used as negative controls in parallel experiments lack neutralizing. Of note, cross-neutralization of the Fab we report is based on only one isolate of each clade and should be confirmed on more isolate per clade in future studies.

In summary, for all three HIV-1 clades tested, the CH1α confers to FabA the capacity to neutralize CD4^+^T-cell infection whereas the corresponding FabG harboring the same paratope but a different CH1, namely CH1γ, has limited neutralizing activities.

#### Blockade of cell-to-cell virus transfer by FabA and FabG

Cell-to-cell transmission of HIV-1 is of major importance *in vivo*, being much more efficient than infection by cell-free virions [29]. At the mucosal level, HIV-1 entry through multilayer mucosal tissues occurs mainly by targeting Langerhans cells (LCs) that in turn, transfer to CD4^+^T-cells [30–33], which constitute the founder-infected cell population. In turn, CD4^+^T-cells migrate out of the mucosal tissue, further spreading the virus. Thus, we evaluated the capacity of both pairs of HESN Fabs to block clades A, B and C virus transfer from LCs to CD4^+^T-cells. Transfer of clade A and C HIV-1 is inhibited by both FabA 43 and 177 in a concentration-dependent manner, as well as that of clade B by FabA 43, inducing a >40% inhibition at 100ng/ml. Transfer of clade B virus remains much less sensitive to FabA 177 (Fig 2C and 2D). FabG 43 blocks transfer of clade A and C viruses but less efficiently than its FabA counterpart (Fig 2C), and FabG 177 was only able to block clade A HIV-1 transfer (Fig 2D). Irrelevant Fab-IgA and Fab-IgG, used as negative controls, fail to interfere with HIV-1 transfer from LCs to CD4^+^T cells.

Of note, taking all the values into consideration (including all viral clades and Fab isotypes) together, at 100ng/ml of Fab, neutralization capacity correlated directly with inhibition of virus transfer (spearman r = 0.6655, p = 0.0219) (Fig 2E).

Taken together, this series of functional analyses demonstrates that switching the CH1α from a mucosal Ab to CH1γ, considerably reduces the Fab antiviral properties, namely both neutralization and HIV-1 transfer from LCs to CD4^+^T-cells (Fig 2F).

### Identification of FabA- and G-specific mimotopes

The molecular basis by which the CH1 region impacts antibody affinity and functions, could be due to selective molecular stringency/flexibility imposed by the CH1 domain on the paratope conformation. Thus, we aimed to determine the conformational epitopes specific for each Fab isotype, namely clones 43- and 177-specific FabA and FabG.

Consequently, a 12 mer random peptide library was used to characterize a set of the best specific peptides, referred to as mimotopes, of both 43 and 177 clones as FabA and FabG using three rounds of successive screening with increasing stringency, as we have previously described [12]. After sequencing 100 phages specific for each of the Fabs, none of the selected mimotopes correspond to linear sequences of gp41, indicating that Fab epitopes are conformational, as already suggested [3]. Whereas a larger set of mimotopes with high occurrence were retrieved on each of the FabA, a limited number of different phage-expressing peptides bound to FabG, in line with the lower affinity of the FabGs compared with FabAs for gp41 (S1 and S2 Tables).

### Conformational FabA and G epitope mapping

The strategy we used to characterize conformational epitopes corresponding to each set of mimotopes, using *in silico* approaches, is based on mimotope mapping onto trimeric gp41 crystal structures, as described in the Material and Methods section and supplementary data. When experimental techniques such as X-ray crystallography of antibody-antigen interactions turn difficult to use [34], such as in the present case, and when the structure of the target is known, *in silico* approaches can provide an appropriate means to identify candidate epitopes to be further tested [35], for instance by competition experiments. Only two gp41 crystal structures of a clade B, but not clade A or C gp41, each in a different conformational state, are available. One structure represents gp41 in the pre-fusion state, together with gp120, although this gp41 lacks the MPER region [36]. The other gp41 structure mimics the 6-Helix bundle state and is composed of three N-helices and three C-helices that form a six helix-bundle for completion of HIV-1 fusion with target cells in a “spring-loaded” model of fusion. In this case, the gp41 construct lacks the gp41 Cys-loop bridging the C and N helices [37].

As Fab 43 is primarily specific for the gp41 MPER [3] and the Cys-loop contains important gp41 epitopes [38], we reconstructed *in silico* the missing parts of clade B gp41 as described in the Material and Methods section using our recently-developed approach [39]. Hence, we first optimized the available crystal structure of gp41 clade B by reconstituting the missing MPER in the pre-fusion structure and the missing loop in the 6-Helix bundle as described in the supplementary data.

Furthermore, as no crystal structures of clade A or C are available in the literature, we constructed clade A and clade C gp41 structures by mapping each of clade A and clade C gp41 sequences onto the clade B gp41 structures, as detailed in the supplementary data. As a result, from these *in silico* modeling calculations, simulation of clade A, B and C gp41 structures in the pre-fusion and 6-Helix bundle conformations were available for epitope mapping studies.

**Fig 3:**
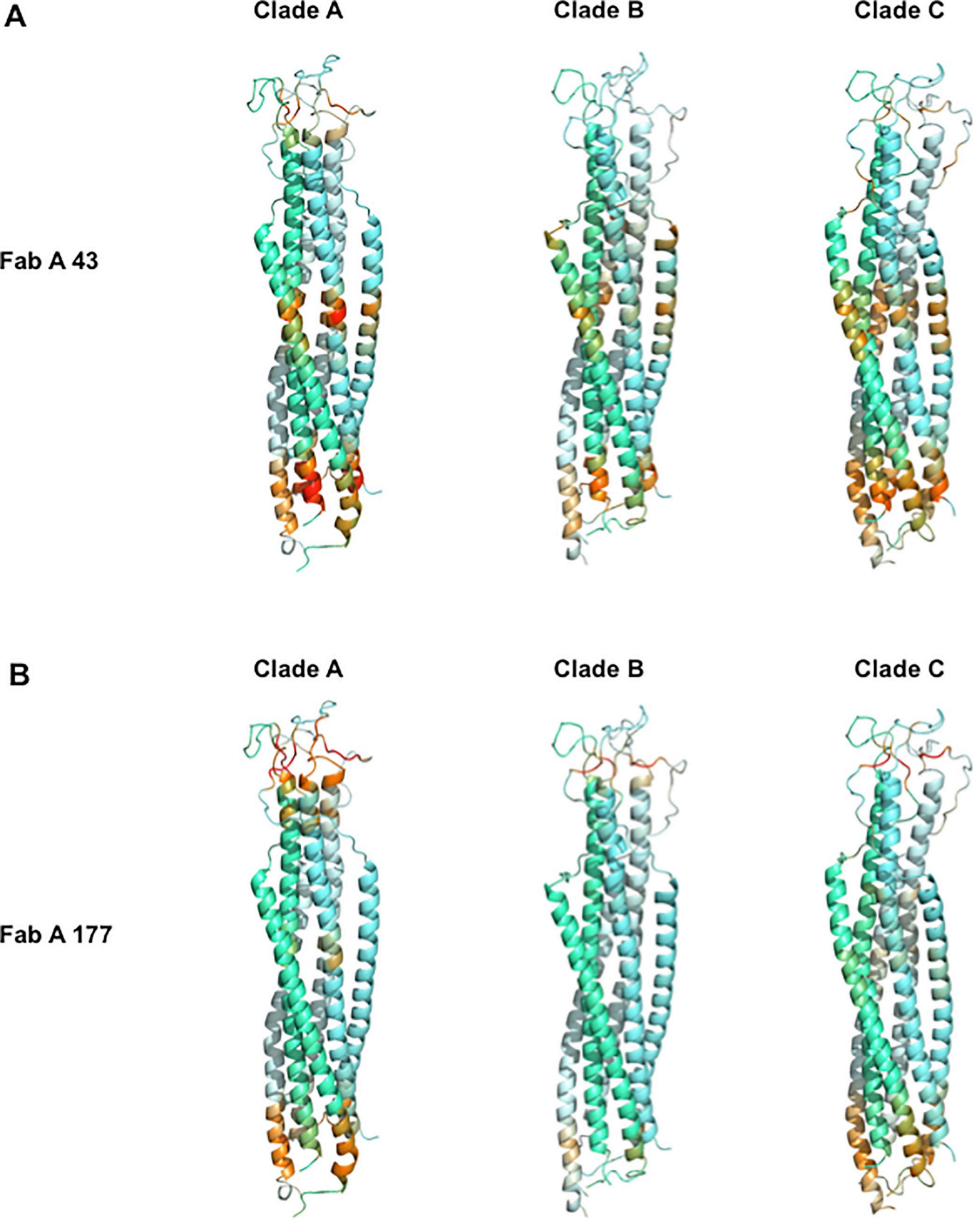
Characterization of epitopes of the FabA clones 43 and 177 on the 6-Helix bundle conformation of HIV-1 envelope gp41. Each set of antibody specific mimotopes obtained by biopanning for both isotype FabA clones 43 (**A**) and 177 **(B**) were docked on the crystal structure of gp41 under its 6-Helix bundle conformation. Each gp41 monomer in the trimer is highlighted by a different hue of blue. Epitopes are visualized in a color scale according to the scores returned by the PEPsurf algorithm at each position of the sequence (mean of the 3 chains, over 15 conformations extracted each 10 ns from the molecular dynamic simulations) for each set of mimotope. A common color scale varying from never (yellow) to the most frequent on all clades and structures (red) is used, allowing to directly compare epitopes on the three gp41 clades. For each score, a set of random sequences at each position of the sequence had been subtracted to remove background noise.

**Fig 4:**
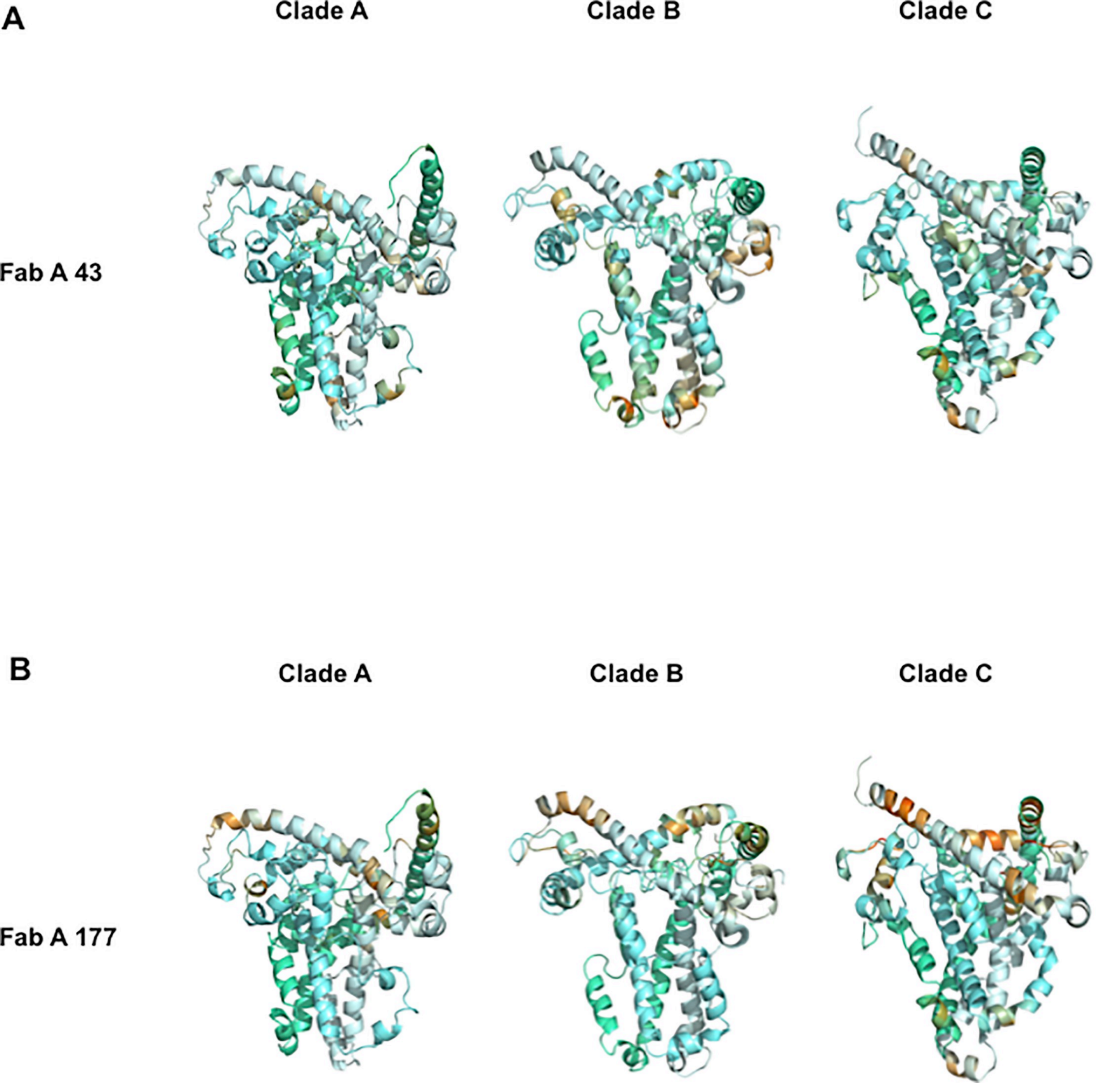
Characterization of epitopes of the FabA clones 43 and 177 on the pre-fusion conformation of HIV-1 envelope gp41. Each set of antibody specific mimotopes obtained by biopanning for both isotype FabA clones 43 (**A**) and 177 (**B**) were docked on the crystal structure of gp41 under its trimeric pre-fusion conformation. Each gp41 monomer in the trimer is highlighted by a different hue of blue. Epitopes are visualized as described in Fig 3 using the same scale to facilitate direct comparison.

Selected FabA-specific mimotopes, obtained from FabA 43 and 177, were localized on clade A, B and C gp41 pre-fusion and 6-helix bundle structures, using the PEPsurf method as detailed in supplementary **S3 Fig**. Two main regions are targeted by FabA 43 mimotopes on gp41 6-helix bundle structure of all three clades, the highest hits being localized on the lower MPER region interface with the N-helix (Fig 3A), in agreement with the screening strategy focusing on P1, used for selecting FabA 43 [3]. In comparison, FabA 43 mimotopes only fit on the clade B pre-fusion structures and with lower scores (Fig 4A). Furthermore, mimotopes fit on each gp41 with slight differences as recapitulated in S3 Table.

The main region targeted by FabA 177 mimotopes on gp41 6-helix bundle structures of all three clades, differs from those targeted by FabA 43 mimotopes, as expected [3]. FabA 177 mimotopes best fit the loop linking the N and C gp41-helices (Fig 3B). These FabA 177-specific mimotopes target an additional domain overlapping the regions mapped by FabA 43 mimotopes, although identified with a much lower score that might not be biologically relevant. Finally, these FabA 177 mimotopes also match gp41 pre-fusion structures as detailed in S2 Table, but with lower scores than the 6-Helix bundle and in a more scattered pattern, irrespective of the clade (Fig 4B) and thus with limited biological relevance.

When FabG sets of specific mimotopes were analyzed similarly, numerous regions were targeted on the various gp41 structures. Corresponding scores might not be relevant biologically, in agreement with the low affinity of FabGs for gp41 and the low number of mimotopes retrieved compared to FabAs. We therefore concentrated our analysis on the FabA 43 and 177 mimotopes.

### Design of peptides recapitulating FabA conformational epitopes

To validate experimentally, the FabA conformational epitopes defined *in silico* as described above, we designed peptides that mimic each conformational FabA epitope and evaluated their capacity to bind their corresponding FabA. To construct these peptides, additional bioinformatic analyses were conducted for each mimotope independently as described in the Material and Methods section.

From these analyses, five peptides specific for FabA 43, namely P7 to P11 could be designed (S4 Table). All of these Fab43 specific peptides appear compatible with the 3-dimensional 6-Helix bundle conformations in all three clades, except P8 which targets only the clade A pre-fusion conformation.

**Fig 5:**
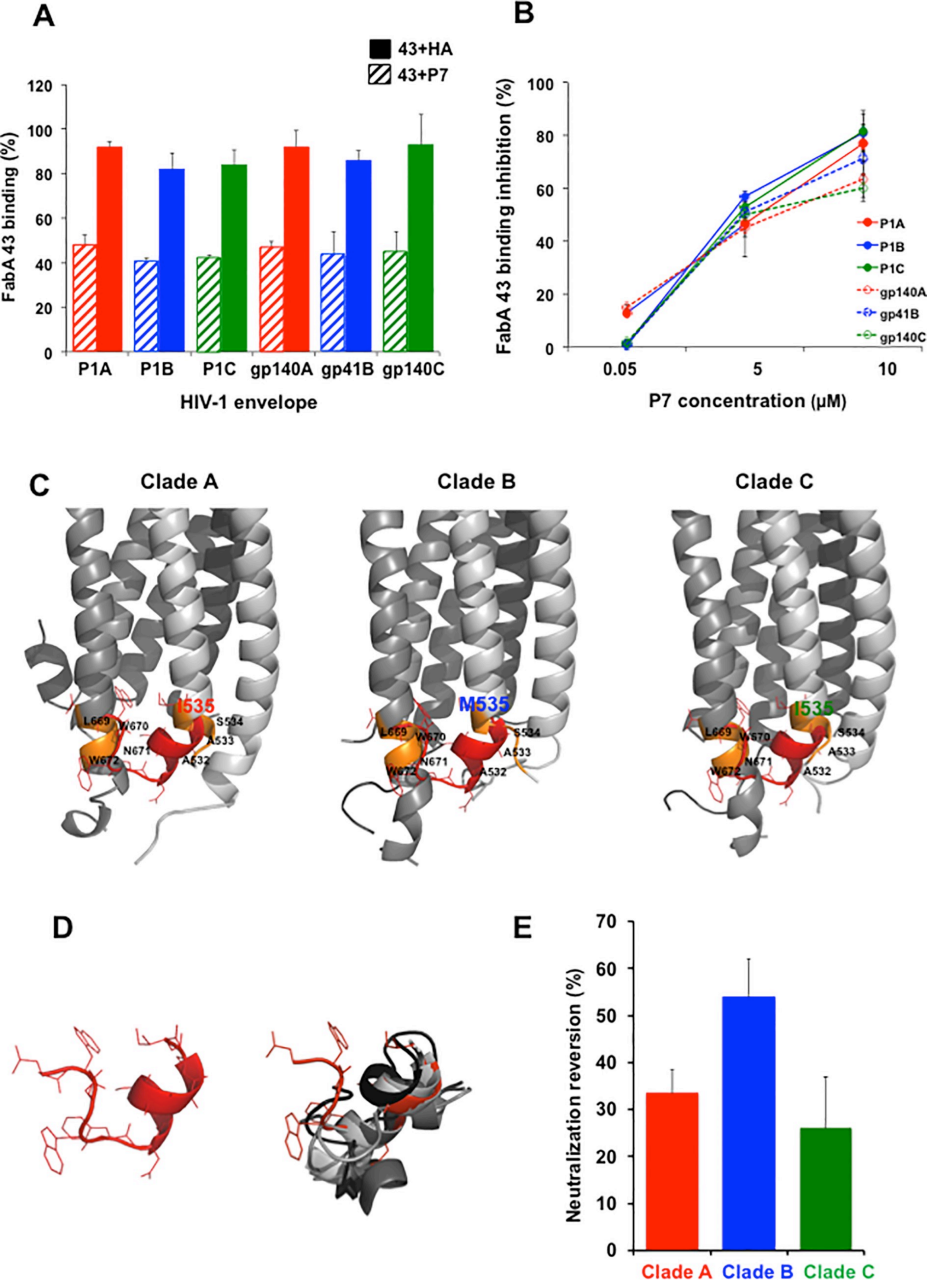
FabA 43 conformational epitope P7 designed *in silico*, blocks FabA 43 binding to gp41 and reduces FabA 43 neutralization activity. **(A)** Preincubation of FabA 43 with the conformational epitope P7 (5 μM, hatch bars) but not with control HA (5μM, plain bars) interferes significantly with FabA 43 binding to clade A (red), clade B (blue) and clade C (green) P1 of clades A and C gp140 and clade B gp41, as evaluated by competition ELISA as described in Material and Method section. FabA 43 preincubated with P7 or control HA were added to gp41 clade B, gp140 (clades A, C) or P1 (clades A, B, C) coated on the ELISA and their binding was detected by using HRP-coupled anti-human IgA. The percentage of binding was calculated relative to the binding of Fab in the absence of the P7 or control HA. (**B)** The P7-induced reduction of FabA 43 binding to each antigen is dose dependent. Competition ELISA was done in the same conditions described in A. Binding inhibition to FabA 43 binding to each antigen in the presence of P7 is calculated relative to the binding inhibition in the presence of the irrelevant HA peptide serving as negative control. In A and B, values represent mean ± SEM, derived from 3 independent experiments performed in triplicate. **(C**) Localization of the amino acid paths corresponding to P7 peptide on the 6-Helix bundle conformation of clades A, B and C gp41 frame15. Each gp41 monomer of the trimer is depicted using a different level of gray. The amino acid paths corresponding to the FaA 43-specific peptide P7 are highlighted in orange. Note residues 532–535 belong to the N-Helix of one monomer while residues 669–672 belong to the C-Helix of another monomer of the trimer. Red: the P7-predicted conformation, superimposed on the structure of gp41. RMSD values over the paired residues of gp41 and P7 are of 2.45, 2.97 and 1.97 Å for clade A, B and C, respectively. Note that in this region of the gp41, only residue 535 differs between clade B in one hand and clade A and C in the other hand. (**D**) Conformational variability of the predicted conformations of P7 using PEP-FOLD3. Left: conformation best fitting gp41. Right: top 10 predicted conformations superimposed. **(E)** Interference of FabA 43 neutralization by a 400-fold molar excess of P7 peptide. FabA 43 preincubated with P7 or control HA were incubated with the HIV-1 of one of each three clades (A, B, C) before adding the activated CD4^+^T cells, and cultures were incubated at 37°C for 2 days. Cultures without Fab were served as positive control of infection. The percentage of neutralization, analyzed by flow cytometry as indicated in Materials and Methods section, is shown relative to FabA 43 neutralization using primary CD4^+^T cells in the presence of the irrelevant HA peptide, tested in parallel. Values represent mean ± SEM, derived from 3 independent experiments performed in triplicate.

When tested at the biological level for FabA specificity, one out of the five conformational epitopes defined *in silico* for FabA 43, encompassing regions on both the gp41 N and C-Helixes with LWNWFDISAASI as sequence, and referred to as P7, competes significantly with FabA 43 binding to P1 and gp41/gp140 of the three clades in ELISA when preincubated with the FabA 43 (Figs 5A and S3A) in a concentration-dependent manner (Fig 5B). P7 at 5 μM blocks FabA 43 binding by >50%, reaching >80% at 10μM (Fig 5B). A 9 amino acid peptide derived from the influenza virus hemagglutinin (HA) used as negative control, does not interfere with FabA 43 binding in ELISA (Fig 5A). In contrast, peptide P7 does not interfere with FabG 43 binding to the same antigens (not shown). Taken together, these results indicate that P7 interferes with FabA 43 cross-clade.

The 12 aminoacid peptide P7 recapitulates a region located at the interface formed by the tips of N- and C-helix of the three clades of gp41 (Fig 5C and 5D). Although P7 has been defined as the best conformational epitope extracted by *in silico* analyses from FabA 43 mimotope fitting to the 6-Helix bundle clade A gp41 structure, this cross-clade reactivity is expected. Hence, the P7- corresponding region in clade B and C is similar in sequence, although it harbors a I535M substitution in clade B. Our predictions support the hypothesis that P7 mimics a patch on gp41 surface involving two different monomers of the gp41 6-Helix bundle, suggesting that FaA 43 could freeze gp41 in the 6-Helix bundle conformation, thereby preventing further conformational changes of the trimer assembly. The other four conformational peptides defined *in silico* for FabA 43 that do not interfere with FabA 43 binding to the gp41 antigens are located on different regions of gp41, regardless of the gp41 clade. As two of them are spatially close from the region defined by P7 (S4 Fig), it suggests that the region mapped by P7 on gp41 is highly specific for FabA 43 binding to the HIV-1 envelope. Furthermore, at the functional level, preincubation of FabA 43 with a 400- fold molar excess of P7 blocked the FabA 43 HIV-1 clades A, B and C neutralizing activities with an efficacy of 33±5, 54±8, and 24±11%, respectively (Fig 5E). These results are in good agreement with the antigen-binding blockade observed in ELISA (Fig 5A and 5B).

Finally, six conformational epitopes, referred to as P0 to P6, that best mimic each set of mimotopes specific for FabA 177, were also designed for each gp41 conformation and clade (S2 and S3 Tables). The six peptides extracted from this analysis are compatible with the 3D conformations of at least two clades and interfere all, although to different degrees, with FabA 177 binding to gp41/gp140 of clades A, B and C, with a maximal binding inhibition limited to 23% (S3B Fig). However, no interference was observed with FabA 177 neutralizing activity against HIV-1 of clades A, B and C.

## Discussion

To decipher the mechanisms of mucosal IgA efficiency in protection against HIV-1 at the molecular level, we investigated whether the CH1 antibody domain can affect the variable region structure, resulting in antibody specificity and affinity changes with direct consequences on Ab functional activities. Using as a model two mucosal IgA derived from HESN individuals that we have characterized previously [3], we now report that FabA, differing exclusively by the CH1 domain from their isotype-switched FabG counterparts, have superior affinity for their specific antigen and higher antiviral activities against representatives of the three main clades of HIV-1, A, B and C, as summarized in Fig 2F.

The present results demonstrate that the CH1 domain influences: (i) antibody affinity, with FabA having a higher affinity to the gp41 subunit antigen, as well as to gp41 within the HIV-1 envelope spike at the surface of HIV-1-infected cells, compared to corresponding FabG (Fig 1 and Table 1), (ii) epitope specificity, since FabA and corresponding FabG recognize different distinct 3-dimensional epitopes on clade B gp41-envelope (S1 Table), although this finding based on *in silico* studies should be confirmed by biological testing. Taken together, these results indicate that the CH1 domain is determinant in dictating the efficacy of the Fab antiviral activities. The FabA, whose CH1 alpha domain increases its affinity for the antigen, efficiently neutralizes CD4^+^T lymphocytes infection and HIV-1 transfer from LCs to autologous CD4^+^T cells, by different representative viral clades distributed worldwide (Fig 2). This contrast with the lower antiviral activities of the corresponding engineered FabG containing a CH1 gamma conferring a reduced affinity for the antigen. Importantly, the CH1 from either IgA or IgG in mammalian gene lacks glycosylation sites [40]. Thus, differences we report here between FabA and FabG, although produced in bacteria, are only dependent on isotype differences.

These differences in antibody affinity, translated into differences in antiviral activities and could result from the differential recognition of multivalent epitope arrays on gp41 envelope, a mechanism proposed to explain the influence of the constant region on antibody paratope [41]. In addition, the CH1-CL and CH1-VH interfaces in the FabA, exhibit a greater rigidity compared to the FabG [15]. Thus, at the molecular level, the CH1 domain appears to exert allosteric effects on the paratope conformation, as suggested by Ofran and coll. [17]. This leads to a differential accessibility of FabA *versus* FabG to gp41 epitopes exposed during binding of the HIV-1 envelope to CD4, its first receptor for infection of target cells, and thus to the recognition of distinctive regions.

We previously isotype-switched a blood-derived neutralizing IgG into an IgA, resulting in an increase in affinity and neutralizing activity in favor of the IgA isotype [12]. Similarly, in the present study, the isotype-switching of a mucosal IgA into an IgG, resulted in a decreased affinity and neutralizing activity. This suggests that the compartment from which the antibody originates, either systemic or mucosal, is not the determinant factor for the CH1 function, although the systemic and mucosal repertoire is highly compartmentalized [42]. The observed superior affinity/function of IgA over IgG could be attributed to the target of the antibodies from both studies, namely gp41. Moreover, these studies demonstrate that the CH1 domain impacts on antibody affinity/functions in the absence of the CH2 and CH3 domains as reported here for FabA/FabG, or in their presence as we, and others, reported for IgG/IgA [12,43].

In addition, biological studies coupled to bio-informatic analyses, have permitted definition of conformational epitopes for both FabA, 43 and 177 on gp41 from clade B but also A and C in two conformational structures of clade-specific gp41, namely pre-fusion and 6-Helix bundle. As available crystal structures of gp41 in these two conformations have only been established for clade B, we reconstructed *in silico*, using our recently developed method [44], clades A and C gp41 in the two conformations, thus offering a useful template for future epitope docking research. The prevailing model of HIV-1 fusion to target cells [45] based on gp41 pre-fusion [36] and 6-Helix bundle [37] crystal structures, allowed localization of these epitopes on each gp41 conformation for clades A, B and C and vulnerability sites targeted by protective HESN IgA to be defined. These sites are preferentially-located on the 6-Helix bundle conformation of gp41, the last step prior to the actual fusion of HIV with the target membrane after dissociation from gp120, whereas location of these epitopes on the pre-fusion structure is less likely.

FabA 43 is an antibody selected based on its specificity for the MPER extended peptide P1 [3]. In the context of the entire gp41 HIV-envelope protein, the main epitope targeted by this FabA 43 on gp41 regions in 6-Helix bundle conformation, is more complex and includes the surface of the N-terminal region of the N-Helix (HR1) of gp41. Further *in silico* studies designed the P7 peptide that recapitulates FabA 43 cross-clade conformational epitopes defined on the 6-Helix bundle of gp41 and comprises both C- and N-Helix facing amino acids. P7 is specific for FabA 43 since it competes for Fab43 A binding to P1 and gp41 of the three A, B and C clades. When in a 400-molar excess, P7 reverses neutralization of HIV from the three HIV-1 clades, albeit with different efficacy. Such clade differences in P7 neutralization reversion correlate with the presence of a Methionine at position 535 in gp41 clade B, whereas it is an Isoleucine in the two other gp41 clades (highlighted in Fig 5C). This position appears therefore, critical for the 3D structure, and the presence of a methionine residue might induce a local conformational change between clade B and clades A and C. The FabA 43 blocking activity of P7 is specific, since other conformational peptides, located in the vicinity of P7, were inefficient in interfering with FabA 43, when evaluated in competition ELISA (S3 Fig).

The P7 sequence, namely LWNWFDISAASI, contains in the very least, part of, if not the entire epitope targeted by two bNAbs IgGs specific for the gp41 MPER region, namely 4E10 specific for NWFDI [46] or 10E8 specific for WNWFDISWLWYIK [21]. Similar to the 10E8 IgG, FabA 43 is encoded by the same VH/DH germline genes [3], namely IGHV3-15*05, IGHD3-3*01, while their JH gene differs, *i*.*e*. IGHJ6*03 for FabA 43 and IGHJ1*01 for 10E8 IgG. In contrast, the 4E10 IgG germline origin, namely IGHV1-69, IGHD3-16, IGHJ1, differs from that of FabA 43. Moreover, CDRH3 loops of FabA 43 (ARDPRYYDAWSGPQLYYYYMDV) and of 10E8 (ARTGKYYDFWSGYPPGEEYFQD) have 86.36% (19 of 22 amino acids) similarity in their sequence, while only 60% (12 of 20 amino acids) match appears between FabA and 4E10 CDRH3 loop (AREGTTGWGWLGKPIGAFAH). Accordingly, FabA 43 does not compete with the 4E10 IgG in binding P1 of clade B, as we reported [3], suggesting that both the antibody origin, mucosal or systemic, and the P7 amino acids located on the gp41 N-terminal, namely ASI, are determinant in the selection of a IGHV3-15 germline.

The present results suggest a molecular basis for an IGHV3-15 gene-biased usage by bNAbs that target a common MPER epitope, such as FaA 43 and 10E8 IgG. It adds to the IGHV1-69 gene-biased usage by the 4E10 IgG targeting also the MPER, and by bNAb IgGs targeting the influenza virus and hepatitis C virus [47]. Additionally, FabA 43 [3] lacks cross reactivity with self-antigens, behaving thus similarly to 10E8 IgG but differently from 4E10 [21]. Whether IGHV3-15 gene, using antibody with affinity for WNWFDI, translates directly into affinity-matured mucosal and systemic MPER-specific antibody response remains to be determined.

Taken together, these analyses indicate that the epitope targeted by HESN mucosal IgA 43 is accessible during the infection process, notably, when gp41 adopts a 6-Helix bundle conformation. A new vaccine, based on the conformational epitopes recognized by these mucosal protective antibodies, such as P7 targeted by FabA 43, could lead to generation of neutralizing antibodies that bind to various regions (epitopes) able to prevent HIV-1 infection.

The contribution of the CH1 domain on epitope specificity provides yet another layer of complexity to the structure-function relationship of anti-HIV-1 antibodies. In particular, the positive impact of CH1α binding to critical regions on the HIV-1 envelope and in virus neutralization, argues in favor of inducing IgA by vaccination. However, current HIV-1 vaccine strategies for preventing mucosal infection focus primarily on the induction of bNAb or variable V1 V2 loop specific IgGs [48], neglecting the IgA response.

HIV-1 vaccines should ideally elicit neutralizing antibodies for protection of mucosal tissues against infection by the various HIV-1 clades worldwide, not only clade B, the best studied virus, but also clade C, endemic to Asia and Africa and clade A, prevalent in Eastern Europe and Africa [49]. Using as vaccine antigen, epitopes of mucosal IgA from HESN with cross-clade anti-viral activities such as P7, might help in the design of a cross-clade vaccine. The design of such a vaccine will require the presentation of the P7 conformational epitope in a formula appropriately immunogenic, administrated by mucosal and systemic routes, to drive the evolution of mucosal FabA 43-like and systemic 10E8-like antibodies.

In summary, the IgA isotype appears to play a major role at the mucosal level, in the protection of HESN individuals because of the influence of the CH1 domain *via* allosteric mechanisms on the paratope conformation. Deciphering the involvement of the CH1 domain in the antibody fine specificity, despite conservation of V region sequence, provides new knowledge, useful for the design of vaccines against HIV-1.

## Material and methods

### Proteins and peptides

The gp41 (584–684) used was a construct based on the HXB2 group M subtype B sequence [50] and kindly provided by Dr. W. Weissenhorn (structural Biology Institute, Grenoble, France). Gp140 from HIV-1 clades A (92RW020) and C (C.ZA.1197MB) were obtained from Immune Technology Corp (NY10021, USA). Sequences of P1 (a.a 630–685) derived from clade B HXB2 gp41 (P1-B) was SQTQQEKNEQELLELDKWASLWNWFDITNWLWYIK as described [19], from clade A 99UGA07072 gp41 (P1-A) was SQIQQKKNEQDLLALDKWANLWNWFDISNWLWYIR and from clade C Bw96Bw0502 (P1-C) was SQTQQEKNEQELLALDSWKNLWNWFSITNWLWYIK. Peptides were chemically synthesized at a purity >95% by Biopeptide (LA, USA) for P1-B and United BioSystems (VA, USA) for P1-A and -C.

### Cloning of FabG and production and purification of soluble FabA and G

HESN FabAs were transformed in their corresponding FabGs by molecular cloning using pASK88 vector which direct the synthesis of the human gamma-1 heavy chain and light chain, respectively [51]. Production of functional Fabs was performed as previously described [3]. Briefly, cultures were grown in 1L of LB medium containing 100μg/ml ampicillin and expression was induced for 14 hr at 22°C by addition of 0.2 mg/L of anhydrotetracycline (ACROS Chimica) at an OD_550_ of 0.5. Fabs were purified from the periplasmic fraction of the E. coli pellet by immobilized metal affinity chromatography (IMAC) using a GE Healthcare kit (GE Healthcare, Sweden, 17-5286-01, HisTrap FF crude). After elution from the affinity column with imidazole 0.5M, the FabA and G were dialyzed against PBS and concentrated using Amicon Ultra-10 centrifugal filter units (Millipore). Antibody purity was evaluated by native SDS/PAGE, followed by Coomassie blue staining resulting in a single band at 50 kDa corresponding to the Fab heterodimer comprising the heavy and light chains, as expected. No band at 25 kDa corresponding to either isolated Fab light or heavy chain could be detected.

The concentration of FabA and G was measured by sandwich ELISA allowing the restricted detection of full Fabs, namely covalently linked Heavy and Light chain heterodimers, using goat anti-human IgA or IgG (Caltag, France) for coating, and biotinylated mouse anti-human Ig kappa light chains (B.D. Pharmingen, USA) for detection, as earlier described [3].

### Binding assays

#### Surface Plasmon Resonance (SPR)

All experiments were performed in duplicate, as described in [12,52] with a Biacore 3000 instrument (Biacore, Inc.) at 20°C in HBS-EP running buffer [10 mM Hepes (pH 7.4), 150 mM NaCl, 3 mM EDTA, 0.005% surfactant P20]. Immobilization of various HIV-1 envelope subunit ligands, namely clade B gp 41, clades A and C gp140, and clades A, B and C P1 peptides, to CM5 chips (GE HealthCare) followed the standard procedures recommended by manufacturer. The final immobilization levels were between 300 and 500 RU to avoid rebinding events, as mentioned in [52]. Initial binding experiments using as analytes clones 43 and 177 of FabA and G, in comparison with non-specific isotypes were performed. No specific signal was observed with non-specific isotypes (recorded value of 0 RU), indicating that the observed binding of Fabs to the different HIV-1 envelope subunits was specific. For kinetic measurements, sensorgrams were obtained by passing various concentrations of the analytes over the ligand surface, at a flow rate of 5μl/min, with a 3-min association phase and a 6-min dissociation phase. The sensor surface was regenerated between each experiment with a single injection of 35 mM NaOH and 1.3 M NaCl at a flow rate of 50 μl/min for 30sec. Identical injections over blank surfaces ran in parallel (and giving a value of 0 RU) were subtracted from the data for kinetic analysis. Binding kinetics were evaluated by linearization using “fit kinetics Langmuir binding type” with BiaEvaluation software (Biacore). Relative Pearson's chi-squared tests assessing goodness of fit were always below 10, which indicated that the models used for fitting adequately describe recorded data.

#### ELISA

ELISA binding assays were performed as described [12] by coating microtiter plates (NUNC-Immuno Plate MaxiSorp Surface, or Peptide Immobilizer Exiqon Peptide Immobilizer, Exiqon) with gp140 (trimeric rgp41 at 0.25 μg/well), P1 (0.1 μg/well), peptides corresponding to conformational epitopes (0.1 μg/well), overnight at 4°C in PBS. Fab binding was detected with a biotinylated mouse anti-human Ig kappa light chains (BD Pharmingen) and streptavidin-HRP or with HRP coupled anti-human IgA (Jackson ImmunoResearch) for the peptides corresponding to conformational epitopes. All experiments were performed with Fabs from at least three independent purifications, each in duplicate. For competition ELISA, Fab 43 was preincubated overnight at 4°C with various concentration of P7 or the irrelevant Hemagglutinin (HA) (YPYDVPDY) peptide serving as negative control, then added to gp41 clade B, gp140 (clades A, C) or P1 (clades A, B, C) coated on the ELISA plate at a final concentration of 0.8nM for FabA 43 and 0.05 to 10μM for P7. Fab binding to each gp41 subunit was finally detected enzymatically with HRP-coupled anti-human IgA (Jackson ImmunoResearch).

#### Fab binding to HIV-1 infected cells

Binding assays were performed as described [53]. For detection of Fab binding to native HIV-1 envelope at the surface of HIV-1-infected cells, FabA or FabG (5μg/ml) were incubated with 10^5^ cells overnight at 4°C. To allow for direct isotype comparison, FabA and G were detected in parallel using the same fluorescein isothiocyanate (FITC)-conjugated mouse anti-human kappa light chain (BD Biosciences, San Jose CA, USA), 30 minutes at 4°C. Cells were fixed with 4% paraformaldehyde, and further stained for intracellular Gag-p24 with the phycoerythrin (PE)-coupled anti-Gag KC57 murine monoclonal antibody (Beckman Coulter GmbH). Binding of irrelevant IgA and IgG to infected cells was set to 1% and subtracted from the specific binding values. Fab binding to target cells was quantified by flow cytometry using a Guava EasyCyte flow cytometer (Merck-Milipore) and analyzed using the dedicated InCyte software.

### Target cells

Peripheral blood samples from healthy donors, obtained from the Etablissement Français de Sang (Paris, France) were depleted of CD8^+^T cells with Rosette Sep cocktail, (StemCell Technologies Inc., France) and peripheral blood mononuclear cells (PBMCs) were isolated by Ficoll-Hypaque. After stimulation for 2 days with 5μg/ml phytohemagglutinin (Sigma-Aldrich, St.Louis, MO) as described [12], CD8-depleted PBMCs were used for infection and neutralization experiments. Alternatively, CD4^+^T CEM-NKR lymphocytic cells (NK-resistant) expressing CCR5 (AIDS Research and Reference Program, NIH) were used when indicated. To prepare CD4^+^ T target cells for neutralization assays, cells were split 1:3 on the day of passage and used the following day.

Primary monocytes were purified from peripheral blood samples from healthy donors using human monocyte enrichment kits (StemCell Technologies Inc., France) and differentiated into Langerhans cell with TGF-β, GM-CSF and IL-4, as described [12].

### Virus stock preparation

A stock of HIV-1_JR-CSF_ (clade B, R5 tropic) was prepared on a large scale by transfecting 293T cells with a plasmid containing the DNA sequence of JR-CSF (NIH, Germantown, MD USA) [3]. The cell culture supernatant was concentrated, separated into single-use aliquots and stored at –80°C.

The HIV-1 primary isolates 92UG031 (clade A, R5) and 92BR025 (clade C, R5), obtained through the NIH AIDS Reagent Program, was amplified on PBMCs, as previously described [3]. Virus concentration was quantified by measuring p24 antigen by ELISA (Innotest HIV-1 Antigen mAb, Innogenetics).

### HIV-1 neutralization assays

#### Single-cycle neutralization assay

The neutralization activity of FabA and FabG was evaluated on primary CD4^+^T-cells, CD8^+^T cell-depleted PBMCs or the CEM-CCR5^+^ cell line infected with HIV-1 JR-CSF (clade B) or with each of clade A or C primary isolates and quantified by flow cytometry after intracellular Gag-p24 staining, as described earlier [3]. At least five independent experiments, performed each in triplicate, were done. Live cells initially gated by forward and side scatter were analyzed for intracellular expression of p24-Ag. A dose-dependent parameter was used to compare the FabA with FabG and for determination of maximum percent inhibition values. Similar concentrations of irrelevant Fab-IgA and Fab-IgG showing a value between 1–10% depending of the clade were used as negative controls. Neutralization was defined as % of cells infected in the absence of antibody. Titers were calculated as IC50 and IC85 and reported as the concentration of Fab causing a 50 or 85% reduction in the percentage of p24^+^ cells compared to virus control wells.

For competing the neutralization, FabA 43 was preincubated with a 400-fold molar excess of P7 overnight at 4°C and further incubated with the virus and the target primary CD4^+^ T-cells at final FabA 43 concentration of 20nM and P7 concentration of 33μM. The irrelevant 9 amino acid peptide from the Hemagglutinin HA (YPYDVPDY) was used as negative control.

#### Inhibition of HIV-1 transfer from cell to cell

The inhibition of HIV-1 transfer from Langerhans cells to T cells was evaluated using monocyte-derived Langerhans cells (LCs) obtained from PBMCs, and autologous CD4^+^T cells as we previously described [12]. Briefly, LCs were incubated with either clade of HIV-1 for 2hrs at 37°C, washed extensively to remove the free virus and distributed in 96 well plates at 100,000 cells / well. Indicated concentrations of FabA or G were then added to the corresponding wells. Finally, either medium alone, or resting CD4^+^T-cells in medium (100,000 cells / well) were added. The LC-T-cell co-cultures were incubated at 37°C for 5 days. Irrelevant Fab-IgA and Fab-IgG at similar concentrations showing a value between 3–7% depending of the clade were used as negative controls. Virus transfer was evaluated by measuring Gag-p24 released in the culture medium using a commercial ELISA (Innotest HIV-1 Antigen mAb, Innogenetics) according to manufacturer instructions. Results are expressed as % inhibition of transfer using formula [(LC+T-cell-Ab)—(LC+T-cell+Ab) / (LC+T-Ab)] x100.

### Epitope mapping

Epitope mapping of both isotypes was performed as we described previously [12], using linear 12-mer peptide libraries displayed on the protein pIII of M13 phages (New England Biolabs) as recommended by the manufacturer. In brief, magnetic beads were incubated with Fab IgA or IgG on a rotating wheel for 2 h at room temperature and epitope screening was initiated by incubating each Fab IgA or IgG coupled beads with the original 12-mer (10^13^) phage-displayed peptide library containing different phages, overnight at 4°C. After three rounds of selection with increasing stringency, the phages were tittered, and single clones were picked and tested by phage ELISA for specific binding on each FabA or FabG. Positive clones were amplified, and each specific peptide insert was sequenced. Importantly, two rounds of negative selection using beads coated with normal human IgA or IgG (Jackson ImmunoResearch) were introduced between two rounds of positive selections. Phages remaining from the negative selection were amplified in *Escherichia coli* ER2738, precipitated, and used for second and third rounds of selection similar to the first round, but with increased buffer stringency as described [12].

### Modeling clade A, B and C gp41

#### Six-Helix bundle clade B gp41

X-ray crystallography studies have been highly successful in giving information about the helical regions of the HIV-1 gp41 ectodomain in its 6-Helix bundle state; however, in all cases to date, the loop region was either removed to facilitate crystallization or not visible in the crystal structure [37,54]. Nevertheless, the structure of the SIV gp41 ectodomain containing the loop region was successfully determined by NMR spectroscopy [55]. The HIV-1 and SIV loops share a high degree of sequence identity (46%). The structure of the trimeric gp41 ectodomain of HIV-1 in its 6-Helix bundle state was therefore built using the X-ray crystallography data available for the 6-Helix bundle of HIV-1, and the NMR data available for the loop of SIV using the MODELLER v9.15 software [56]. The conserved disulfide bond of the loop between residues C68 (C599) and C74 (C605) was added as a constraint. This bond was proved to be critical to the furin recognition site of HIV-1 gp160 [57].

The crystal structure of the 6-Helix bundle HIV-1 gp41, including both fusion peptide and membrane proximal external regions [37], was retrieved from the RCSB Protein Data Bank (http://www.rcsb.org, code: 2X7R). The solution NMR structure of ectodomain of SIV gp41 [58] was retrieved from the RCSB Protein Data Bank (http://www.rcsb.org, code: 2EZO). Due to the high sequence similarity, the multiple sequence alignment used to build the 6-Helix bundle structure of HIV-1 gp41 was generated using T-Coffee [50] (S4 Fig).

#### Pre fusion clade B gp41

The structure of the trimeric gp41 ectodomain of HIV-1 in its pre-fusion state was built using the only available crystallographic structure of the pre-fusion HIV-1 gp41(33) (http://www.rcsb.org, code: 4TVP). Missing loops were modeled using DaReUS-Loop which identifies loop candidates in large collections of structures using a new similarity approach based on a Binet Cauchy (BC) kernel [39].

#### Pre fusion and 6-Helix bundle clades A and C gp41

The clade A and clade C gp41 sequences were retrieved from http://www.hiv.lanl.gov (code: AF484478 and code: AF110967, respectively) aligned (S5 and S6 Figs) and mapped onto the structures modeled for clade B using the MODELLER v9.15 software [56].

We used all atom molecular dynamic (MD) simulations to inspect the behavior of the 6 trimeric structures (pre-fusion: clades A, B and C, 6-Helix bundle: clades A, B and C) and explore their conformational space. The simulations were performed under periodic boundary conditions by defining a box with a minimal distance of 10.0 Angströms between the protein and the wall of the cell. The box was then filled with water and we added a concentration of 0,2 M Na^+^Cl^-^ to the solvent, as well as an excess of Na^+^ ions to neutralize the system charge induced by the negatively charged residues. 200 ns of Molecular Dynamic simulations were then run for the structures using Gromacs 2016.1 [59,60] with AMBER99SB-ILDN [61] as force field and TIP3P [62] model for water, using virtual sites to allow a 5 fs integration step.

### Mimotopes sequence analyses and mapping on gp41 models to define antibody binding sites

Epitope mapping on pre-fusion and 6-Helix bundle clades A, B and C gp41 was performed for each set of FabA and FabG mimotopes, using PEPsurf [63]. This was done as we described previously to characterize the canonical epitope of the 2F5 antibody, as well as additional discontinuous 3D epitopes from a set of mimotopes [12]. Hence, PEPsurf determines 3D epitopes on the target protein surface by searching paths for a 3D fit with partial amino acid strings of a given sequence in a preset distance. PEPsurf compares each peptide provided with the solvent accessible surface of the antigen, determining the best paths. Taking into account the frequency of each mimotope, the algorithm then creates clusters of antigen residues on the surface that best fit a group of paths and returns for each residue, a score corresponding to the propensity of the amino acid to be traversed by the paths.

To take into account structural fluctuations, snapshots of the gp41 trimeric structures were taken every 10 ns of the MD simulation, starting from 60 ns (15 frames, where frame 1 corresponds to the conformation at 60 ns of simulation, and frame 15 to the conformation at the end of the simulation). These were explored using the PEPsurf algorithm with both BLOSUM62 and BLOSUM80 as similarity matrix and each set of mimotopes specific of each Fab (34 for 43A, 50 for 177A) as input, independently. Only minute differences were observed by using either BLOSUM62 or BLOSUM80, and the results obtained correspond to those obtained with BLOSUM62. Scores were then averaged for each residue position over the 15 frames. To account for the background noise, a library of 147 12-mer soluble peptides was generated using the SolyPep facility available on the RPBS web portal (http://bioserv.rpbs.univ-paris-diderot.fr/services/SolyPep/). Scores obtained for the random peptide library were then subtracted to the scores obtained with the mimotopes.

Using this method, we found for each antibody, several regions that could correspond to conformational 3D epitopes on the 6-Helix bundle structures of the 3 clades, as shown in Fig 3 and S3 Table.

The Fab 43A-specific mimotopes map to main regions, namely G531-Q540 and E/A662-F673, which correspond to the N-terminus of the N-Helix and the C-terminus of the C-Helix of each monomer, respectively, and E560-T569 and I635-I/L646, which are roughly located in the middle of both helices an epitope that may become closer to the MPER region during conformational changes occurring in gp41 during the spring-loaded model of fusion [37]. The Fab 177A-specific mimotopes map mainly to the regions at the tip of gp41, namely Q590-W596 and A/N607-A/S613, although on clades A and C, the gp41 middle region (E560-T569 and I635-I/L646) was also mapped.

Results for the pre-fusion structures of all clades were less clear (Fig 4 and S3 Table), but highlighted amino acids, which included the P1 region (A/E662-F673) and another near the epitope found in the 6-Helix bundle structure at A/N607-A/S613.

### Identification of candidate epitope-specific peptides

Although PepSurf considers the relative distance and physicochemical properties of individual amino acids, it does not take into account the 3D conformation that the mimotopes might adopt in the solvent. Each of the mimotopes were thus modeled independently, using the PEP-FOLD3 algorithm, a *de novo* approach aimed at predicting peptide structures from amino acid sequences [64]. The 10 best predicted peptide conformations of each mimotope were then structurally aligned with the paths found by the PepSurf algorithm on each of the 15 MD frames of each of the six modeled structures of gp41 (clades A, B and C gp41 in pre-fusion and 6-Helix bundle conformations) to identify paths (i) involving gp41 residues distant in the sequence (*i*.*e*. conformational epitopes), and (ii) meeting the condition of a 3D superimposition onto gp41 structure with a Root Mean Square Deviation (RMSD) lower than 3 Angströms for at least one conformation with one of the 15 frames of gp41. Note, that identified paths proposed by PepSurf do not always encompass the full set of 12 amino acids of the mimotopes. These conformational paths consisting in non-contiguous amino acids of gp41 were, in turn, modeled with PEP-FOLD3, and selection was applied using a similar procedure. Additional manual sequence design (residue ordering, insertion/deletion and substitution) was performed to best fit the three clades together for each peptide. In practice, such design was only successful for P7. The identified sequences were evaluated for their propensity to aggregate in an aqueous solvent using the Waltz server [65].

We illustrate this methodology for P7. S5 Fig depicts the initial matches identified by PepSurf for the mimotope of the sequence NHIPGQPASIFS, precursor of P7. Only 9 positions over 12 were calculated to be matching. They correspond to the sequence NNIGSASIF that encompass, in an un-ordered manner, positions 668–676 (seg1) on the C-Helix of one monomer and 531–535 (seg2) on the N-Helix of another monomer. For this path, the RMSD condition was satisfied for only clade A frame 1. Further design consisted in re-considering the order of the amino acids so as to mimic the assembly of seg1 and seg2 (*i*.*e*. maximizing the similarity with gp41), while meeting the RMSD constraint for, if possible, a larger number of frames of clades A, B and C. This led to the identification of the LWNWFDISAASI sequence of the P7 peptide where -A-SI corresponds to three residues located at gp41 Nter belonging to seg2, -N-F-I-S corresponds to four residues Cter belonging to seg1. G531 was discarded as well as N668, and the addition of another alanine (equivalent to A535) was necessary to obtain a satisfactory superimposition onto gp41. Of note, compared to the original mimotope, no prolines were retained. From this, the 3D linker between the two helices of gp41 located on chains A and B was found to correspond to FDIS. In the end, P7 was found to meet the RMSD constraint for 13, 12 and 12 frames over 15 for clades A, B and C, respectively, excluding the residues of the linker.

### Statistical analysis

Statistical significance was analyzed by the two-tailed Student’s t-test and multiple comparisons were performed using the non-parametric Mann-Whitney U test using Prism 5 (GraphPad, San Diego, CA) software. Heatmap was established using the heatmapper web site (http://www.heatmapper.ca/expression/) using the complete linkage clustering method and non-parametric spearman correlations as distance measurement method. *P values* <0.05 were considered significant.

## Supporting information

S1 Fig Comparative affinities of A and G istotype of Fab 43 and 177 to clades A, and C gp140 and to clade B gp41. Recombinant clades A (**A**), and C (**C**) gp140 and clade B gp41 (**B**) were each immobilized on a CM-5 chip for surface plasmon resonance evaluation of antibody affinity constant. Fab 43A, 43G, 177 A or 177G at the indicated concentrations were the analytes. The KD and corresponding Pearson’s Chi2 test (Chi2) values shown were estimated by global curve fitting of the specific binding responses. Fitted curves are in different colors corresponding to increasing analyte concentration, as indicated below each graph. Injections were carried out in duplicates and gave essentially the same results. Only one of the triplicates is shown.
(TIFF)

S2 Fig Comparative binding of Fab 43 used as analyte to clades A, B and C P1. P1 from A, B and C clades were each immobilized on an independent channel of the same chip. Binding of Fab 43 to the three P1 clades was measured simultaneously to allow direct comparison. FabA was injected at 20nM concentration (**A**), whereas FabG 43 concentration was 100nM (**B**). Graphics are representative of n = 3 independent experiments.
(TIFF)

S3 Fig **Interference of conformational epitopes designed in silico from FabA with FabA binding to gp41 clades A, B and C. A:** in silico epitopes P7 to P11 designed from FabA 43. **B:** in silico epitopes P1 to P6 designed from FabA 177. FabA 43 (**A**) or 177 (**B**) was preincubated with conformational epitopes P7 to P11 (**A**) or P1 to P6 (**B**) or HA peptide used as negative control (all at 5 μM) and further used to detect FabA binding to their respective antigens from clades A, B, C by ELISA. Binding inhibition is shown relative to FabA binding inhibition to each antigen in the presence of HA peptide control.
(TIFF)

S4 Fig FabA 43 conformational epitopes P8-P11 designed in silico, do not block FabA 43 binding to gp41. Localization of the amino acid paths corresponding to P8-P11 peptides on the pre-fusion conformation of clade A gp41 for P8, and on the 6-Helix bundle conformations of clade B for P9, clade C for P10 and P11, i.e. the conformation/clade from which the peptides have been identified. Each gp41 monomer of the trimer is depicted using a different tone of gray. The amino acid paths corresponding to the FabA 43-specific peptides are highlighted in red. Note that P9 and P10 involve amino acids belonging to different monomers of the 6-Helix bundle trimer, while P8 and P11 involve amino acids belonging to the same monomer.
(TIFF)

S5 Fig Multiple sequence alignments used for the initial identification by PepSurf of matches to determine the precursor of P7. 2 Matching positions on indicated gp41 trimer sequences were calculated and localized on the C-Helix of one monomer and N-Helix of another monomer. The alignment of only one monomer is shown.
(TIFF)

S6 Fig Multiple sequence alignment of the three clades of HIV-1. The alignment of only one monomer is shown. 1
(TIFF)

S7 Fig A: Initial path identified on the surface of gp41 clade A by PEPsurf for the 43 IgA mimotope precursor of P7. Only 9 amino-acids match, and span positions 531–535 on monomer 2 and 668–676 on monomer 1. The mimotope sequence is aligned with the following successive positions (from 1 to 9): 1: N668 (1), 2: N671 (1), 3: I675(1), 4: G531(2), 5: S676 (1), 6: A532 (2), 7: S534 (2), 8: I535 (2) et 9: F673 (1), where (1) and (2) denote the monomers a and b of the trimer. B: Structural superimposition of the mimotope, gp41 matching peptide onto gp41 clade A. Green: mimotope of 43 IgA (NHIPGQPASIFS) modeled with PEP-FOLD (RMSD: 2,51Å) Orange: gp41 amino acids corresponding to the path found by PEPsurf; Arrows indicate the match order. Red: path modeled with PEP-FOLD (NNIGSASIF) (RMSD: 2,75Å).
(TIFF)

S1 Table Mimotope sequences specific for 43 and 177 FabA clones. Mimotopes of clone FabA 43 and FabA 177 were obtained by individual screening of a 12 random peptide library expressed on phages as described (12). The precursor mimotopes of several peptides, from P0 to P11 (see S3 Table) are identified. Frequency indicates the times precursor mimotope were found during the screening.
(TIFF)

S2 Table Mimotope sequences specific for 43 and 177 FabG clones. Mimotopes of clone FabG 43 and FabG 177 were obtained by individual screening of a 12-mer random peptide library expressed on phages as described (12). Frequency indicates the times precursor mimotope were found during the screening.
(TIFF)

S3 Table Epitope sequences specific for 43 and 177 FabA clones. Epitopes are derived from in silico analysis of each set of FabA 43- and 177-specific mimotopes on pre-fusion and 6-Helix bundle gp41 structures. Amino acid numbers correspond to numbering of the full HIV envelope. Only regions with a PEPsurf score of more than 0.2 and longer than 2 residues are considered.
(TIFF)

S4 Table **Sequences of candidate conformational epitopes on gp41 Clade A, B and C specific for 43 and 177 FabA clone** Conformational epitopes were obtained by docking each set of specific mimotopes on the gp41 as detailed in the Method section. The clade (A, B, C) from which the precursor was identified is reported, together with the conformation of gp41—pre (Pre) and 6-Helix bundle (6-H). The number of frames (over 15) for which of the 3D condition is satisfied is given for each clade (3D-hits). Cross M.: peptide predicted to mimic a patch involving only one monomer (-) or two monomers of the trimer (+).
(TIFF)
