# Supplementary figures and images for: The CH1α domain of mucosal gp41 IgA contributes to antibody specificity and antiviral functions in HIV-1 highly exposed Sero-Negative individuals
Source: PLoS Pathog. 2020 Dec 14;16(12):e1009103. doi: 10.1371/journal.ppat.1009103 (PMC7802955; doi:10.1371/journal.ppat.1009103)

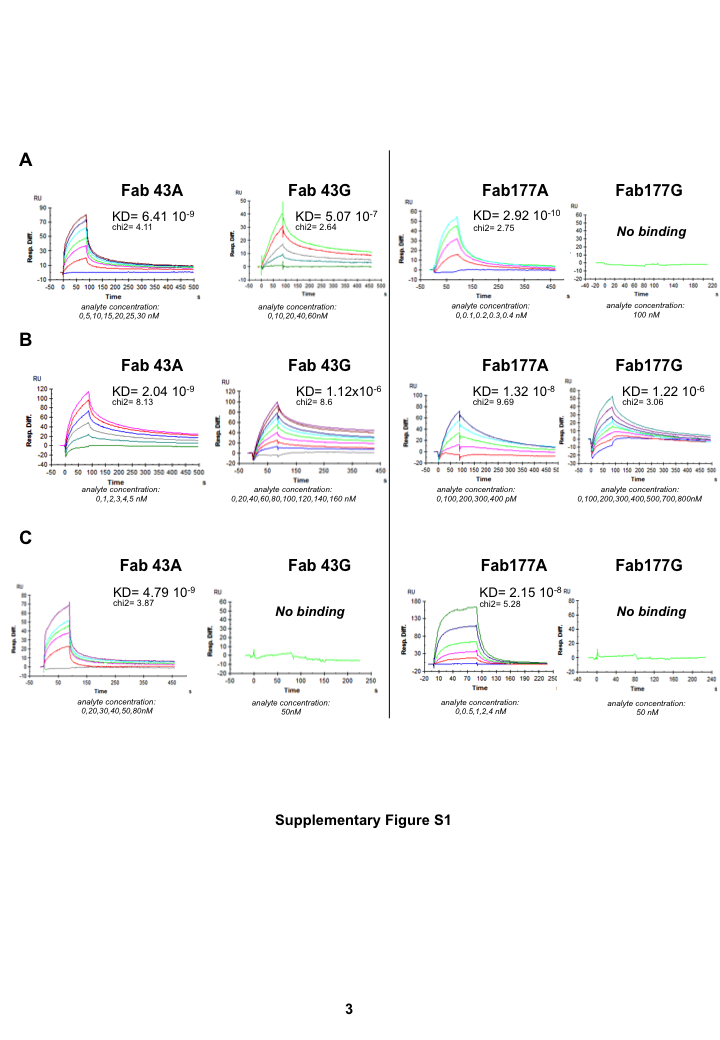

Supplement: S1 Fig — Recombinant clades A (A), and C (C) gp140 and clade B gp41 (B) were each immobilized on a CM-5 chip for surface plasmon resonance evaluation of antibody affinity constant. Fab 43A, 43G, 177 A or 177G at the indicated concentrations were the analytes. The KD and corresponding Pearson’s Chi2 test (Chi2) values shown were estimated by global curve fitting of the specific binding responses. Fitted curves are in different colors corresponding to increasing analyte concentration, as indicated below each graph. Injections were carried out in duplicates and gave essentially the same results. Only one of the triplicates is shown. (TIFF) [file ppat.1009103.s001.tiff]

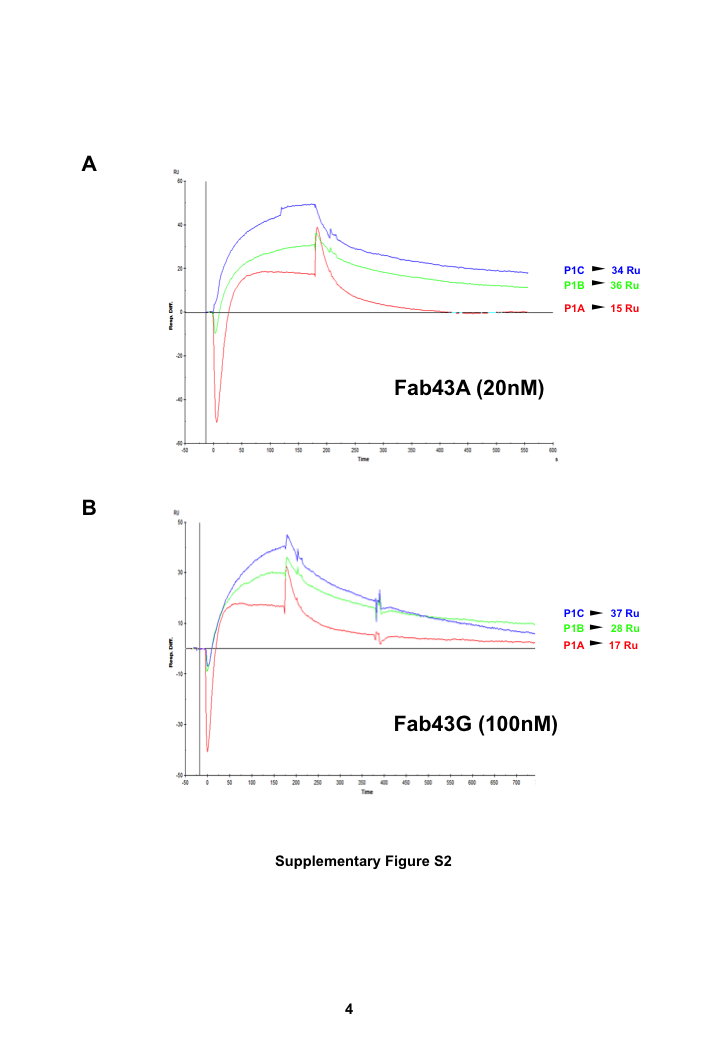

Supplement: S2 Fig — P1 from A, B and C clades were each immobilized on an independent channel of the same chip. Binding of Fab 43 to the three P1 clades was measured simultaneously to allow direct comparison. FabA was injected at 20nM concentration (A), whereas FabG 43 concentration was 100nM (B). Graphics are representative of n = 3 independent experiments. (TIFF) [file ppat.1009103.s002.tiff]

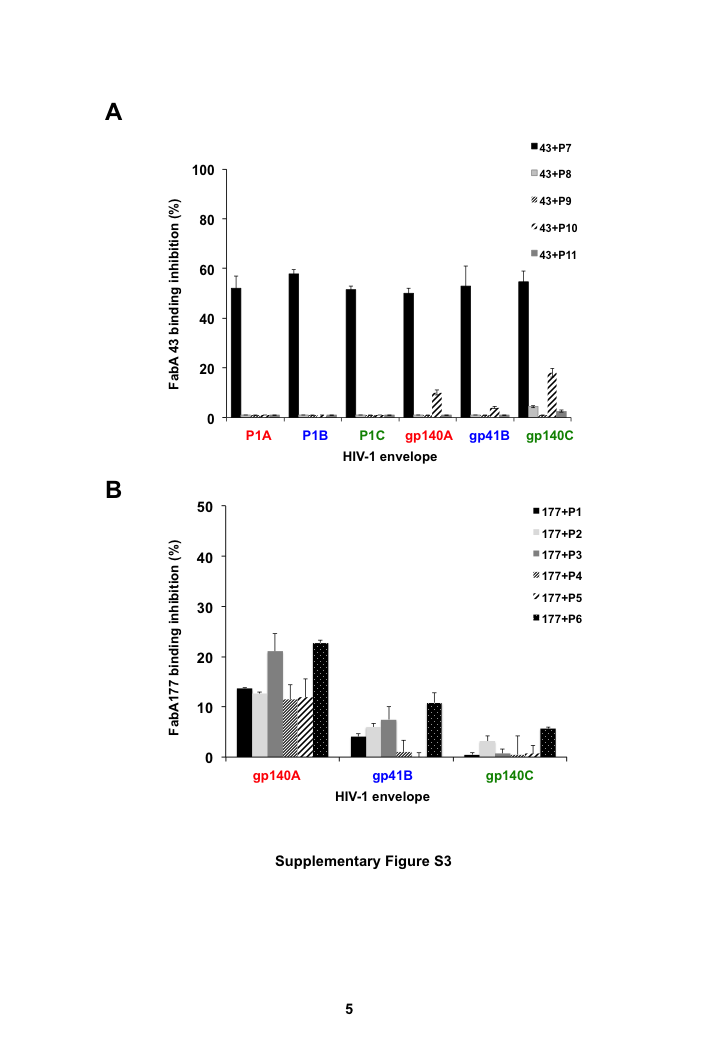

Supplement: S3 Fig — Interference of conformational epitopes designed in silico from FabA with FabA binding to gp41 clades A, B and C. A: in silico epitopes P7 to P11 designed from FabA 43. B: in silico epitopes P1 to P6 designed from FabA 177. FabA 43 (A) or 177 (B) was preincubated with conformational epitopes P7 to P11 (A) or P1 to P6 (B) or HA peptide used as negative control (all at 5 μM) and further used to detect FabA binding to their respective antigens from clades A, B, C by ELISA. Binding inhibition is shown relative to FabA binding inhibition to each antigen in the presence of HA peptide control. (TIFF) [file ppat.1009103.s003.tiff]

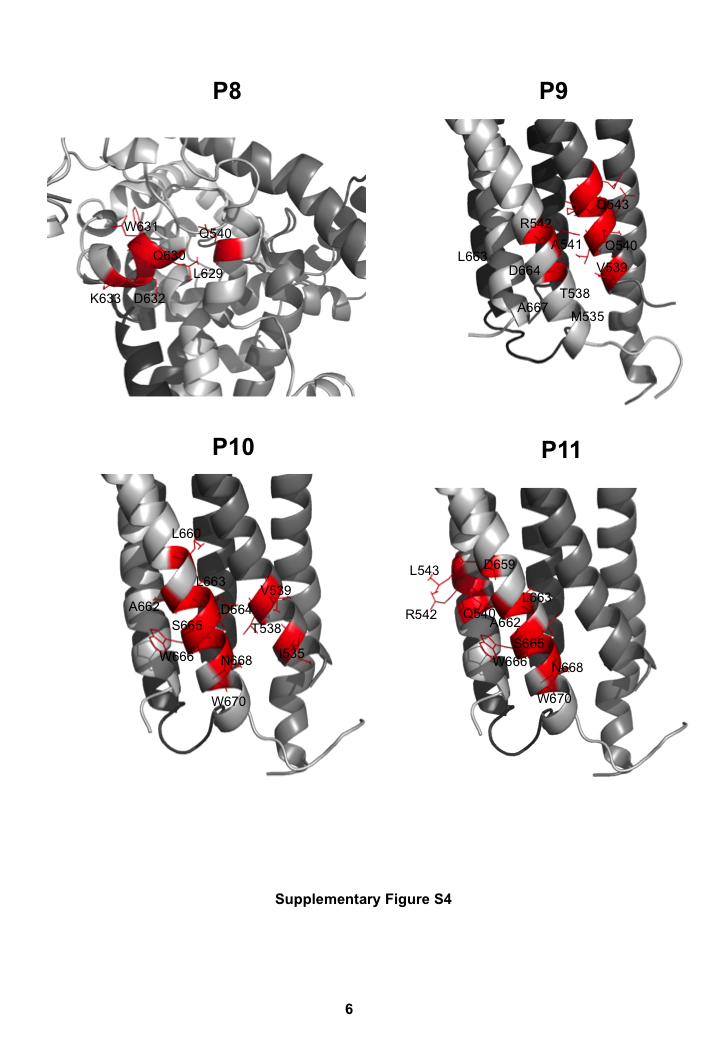

Supplement: S4 Fig — Localization of the amino acid paths corresponding to P8-P11 peptides on the pre-fusion conformation of clade A gp41 for P8, and on the 6-Helix bundle conformations of clade B for P9, clade C for P10 and P11, i.e. the conformation/clade from which the peptides have been identified. Each gp41 monomer of the trimer is depicted using a different tone of gray. The amino acid paths corresponding to the FabA 43-specific peptides are highlighted in red. Note that P9 and P10 involve amino acids belonging to different monomers of the 6-Helix bundle trimer, while P8 and P11 involve amino acids belonging to the same monomer. (TIFF) [file ppat.1009103.s004.tiff]

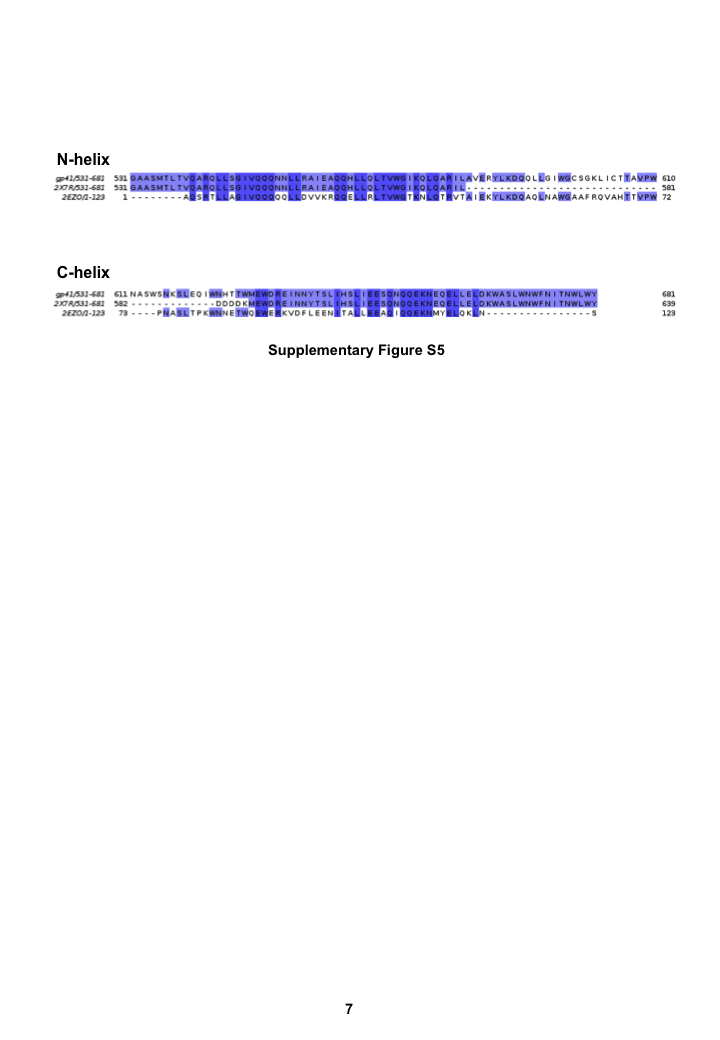

Supplement: S5 Fig — 2 Matching positions on indicated gp41 trimer sequences were calculated and localized on the C-Helix of one monomer and N-Helix of another monomer. The alignment of only one monomer is shown. (TIFF) [file ppat.1009103.s005.tiff]

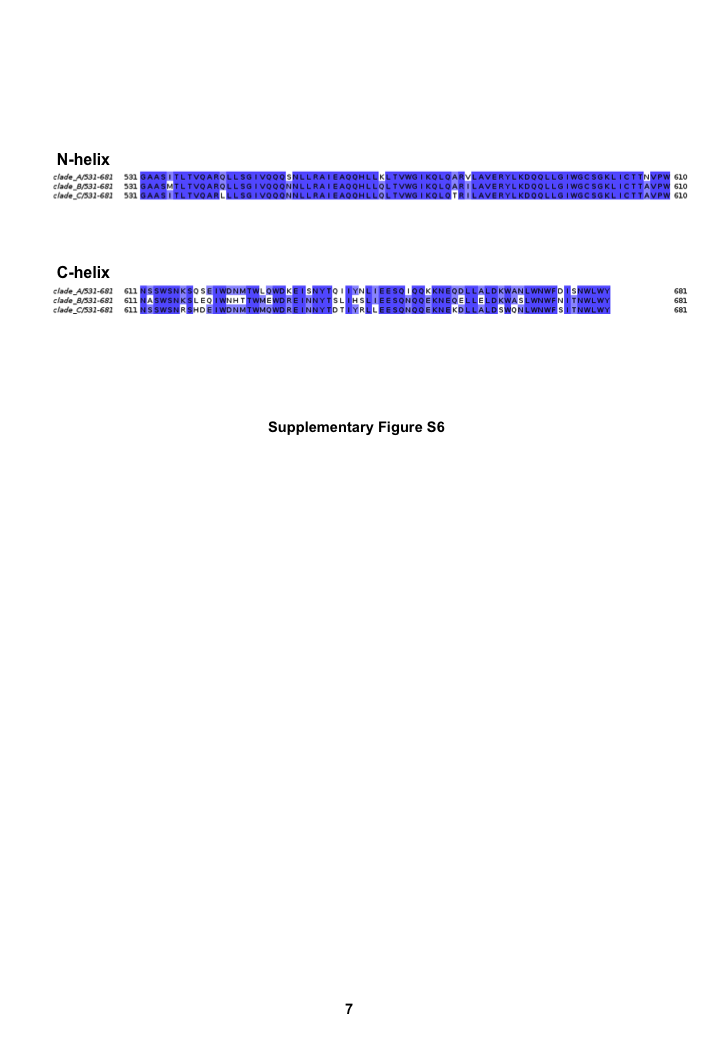

Supplement: S6 Fig — The alignment of only one monomer is shown. 1 (TIFF) [file ppat.1009103.s006.tiff]

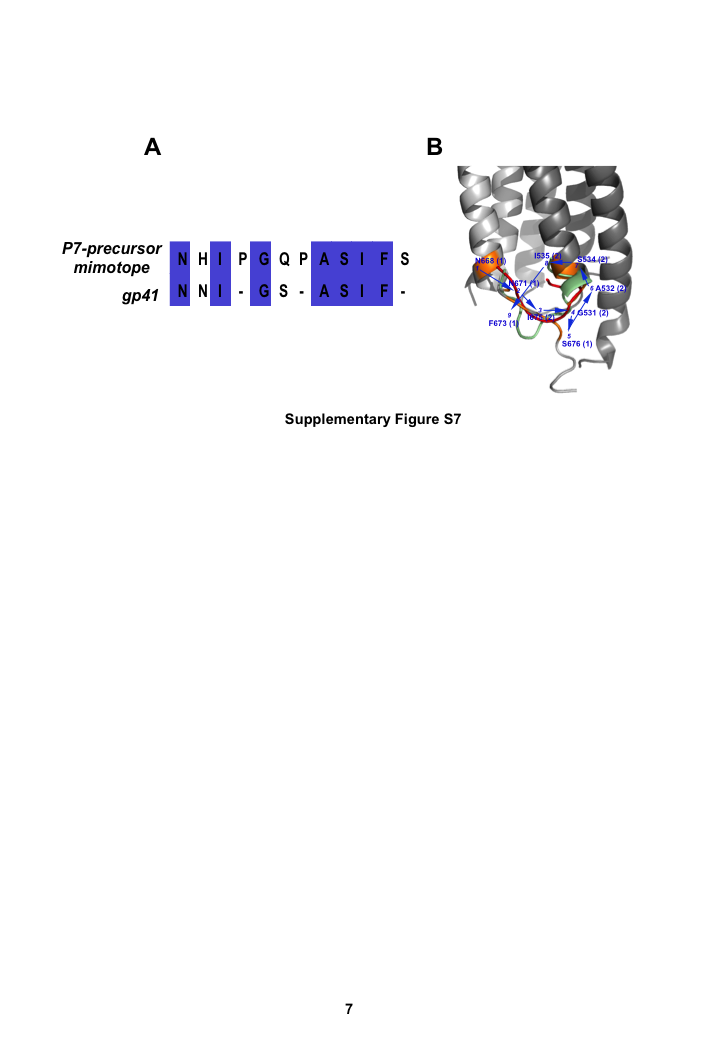

Supplement: S7 Fig — A: Initial path identified on the surface of gp41 clade A by PEPsurf for the 43 IgA mimotope precursor of P7. Only 9 amino-acids match, and span positions 531–535 on monomer 2 and 668–676 on monomer 1. The mimotope sequence is aligned with the following successive positions (from 1 to 9): 1: N668 (1), 2: N671 (1), 3: I675(1), 4: G531(2), 5: S676 (1), 6: A532 (2), 7: S534 (2), 8: I535 (2) et 9: F673 (1), where (1) and (2) denote the monomers a and b of the trimer. B: Structural superimposition of the mimotope, gp41 matching peptide onto gp41 clade A. Green: mimotope of 43 IgA (NHIPGQPASIFS) modeled with PEP-FOLD (RMSD: 2,51Å) Orange: gp41 amino acids corresponding to the path found by PEPsurf; Arrows indicate the match order. Red: path modeled with PEP-FOLD (NNIGSASIF) (RMSD: 2,75Å). (TIFF) [file ppat.1009103.s007.tiff]

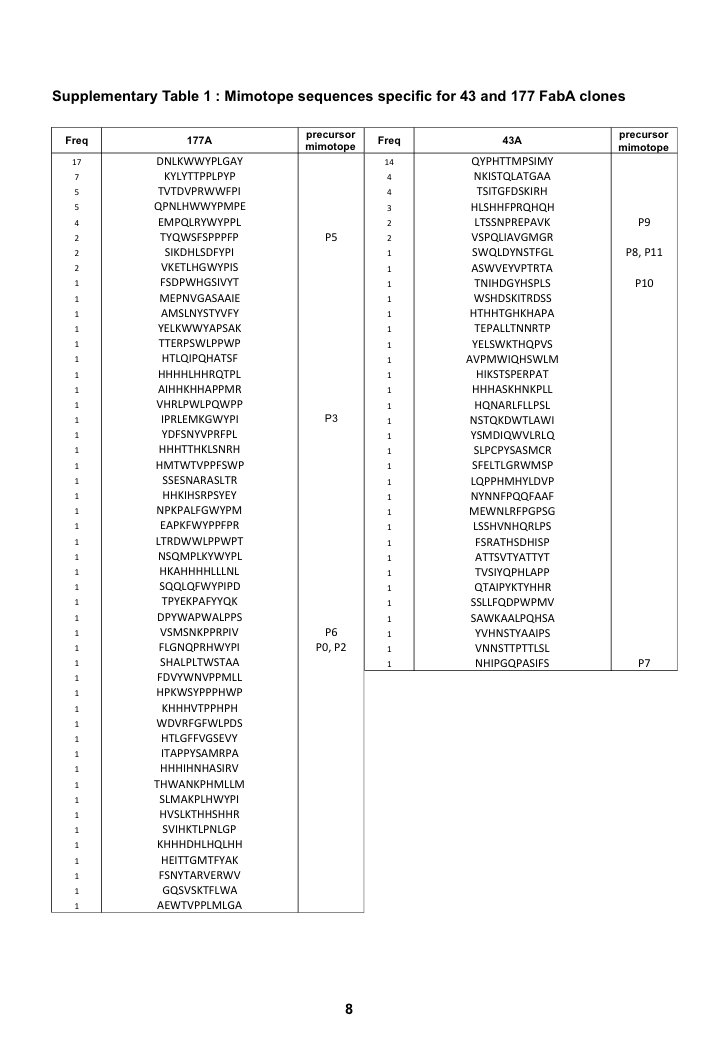

Supplement: S1 Table — Mimotopes of clone FabA 43 and FabA 177 were obtained by individual screening of a 12 random peptide library expressed on phages as described (12). The precursor mimotopes of several peptides, from P0 to P11 (see S3 Table) are identified. Frequency indicates the times precursor mimotope were found during the screening. (TIFF) [file ppat.1009103.s008.tiff]

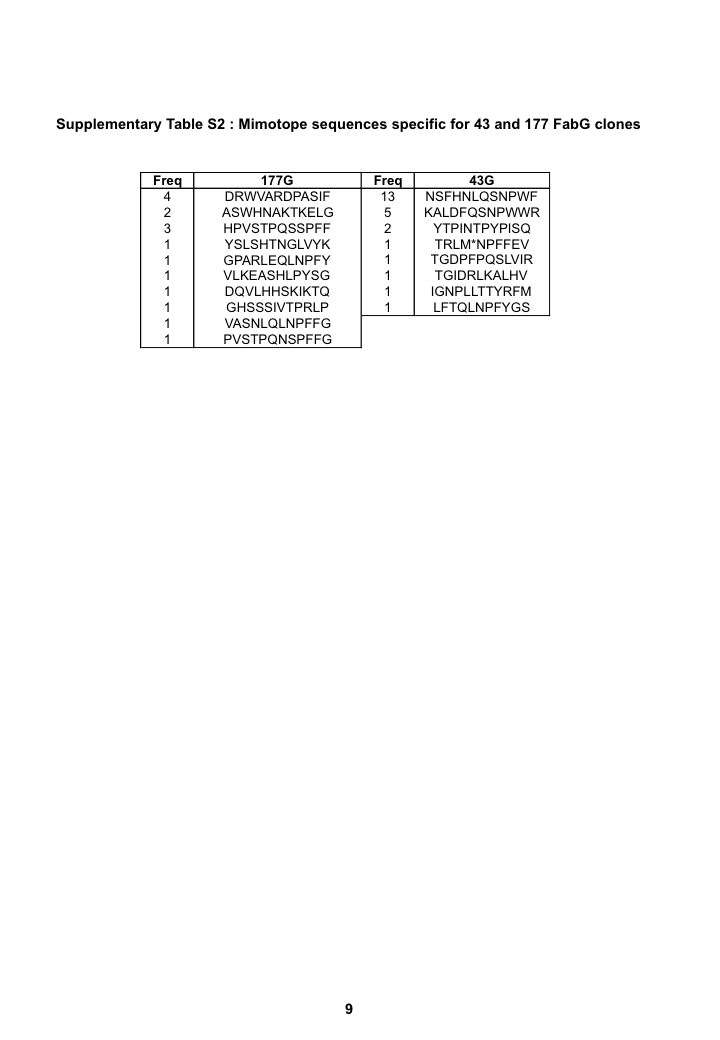

Supplement: S2 Table — Mimotopes of clone FabG 43 and FabG 177 were obtained by individual screening of a 12-mer random peptide library expressed on phages as described (12). Frequency indicates the times precursor mimotope were found during the screening. (TIFF) [file ppat.1009103.s009.tiff]

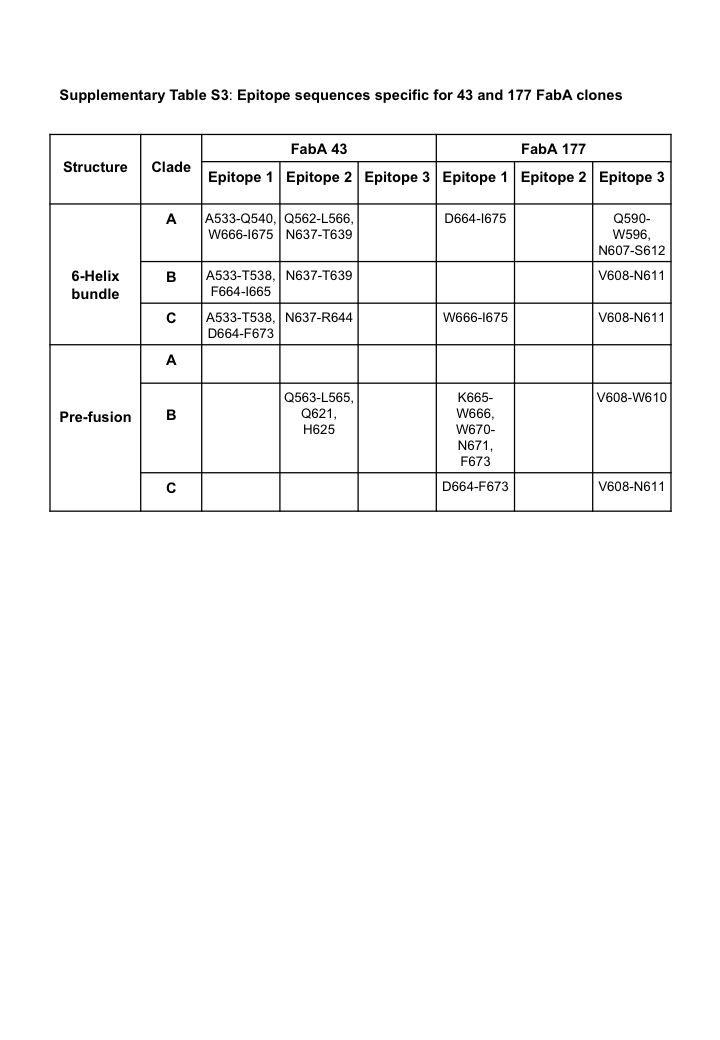

Supplement: S3 Table — Epitopes are derived from in silico analysis of each set of FabA 43- and 177-specific mimotopes on pre-fusion and 6-Helix bundle gp41 structures. Amino acid numbers correspond to numbering of the full HIV envelope. Only regions with a PEPsurf score of more than 0.2 and longer than 2 residues are considered. (TIFF) [file ppat.1009103.s010.tiff]

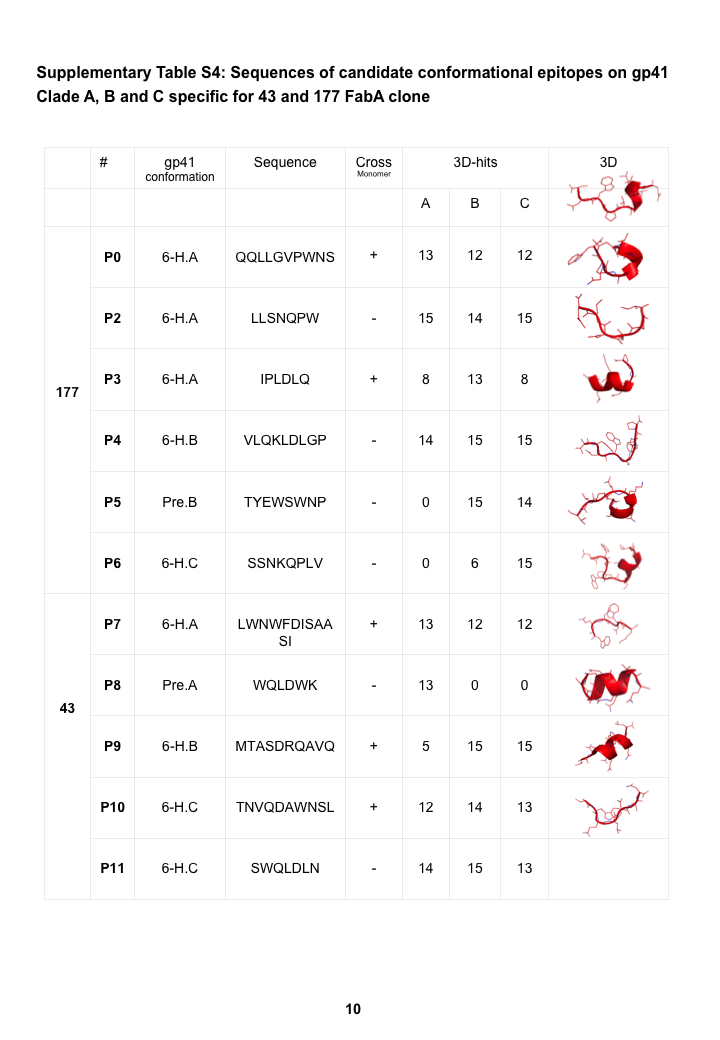

Supplement: S4 Table — Sequences of candidate conformational epitopes on gp41 Clade A, B and C specific for 43 and 177 FabA clone Conformational epitopes were obtained by docking each set of specific mimotopes on the gp41 as detailed in the Method section. The clade (A, B, C) from which the precursor was identified is reported, together with the conformation of gp41—pre (Pre) and 6-Helix bundle (6-H). The number of frames (over 15) for which of the 3D condition is satisfied is given for each clade (3D-hits). Cross M.: peptide predicted to mimic a patch involving only one monomer (-) or two monomers of the trimer (+). (TIFF) [file ppat.1009103.s011.tiff]
